# Learning molecular dynamics with simple language model built upon long short-term memory neural network

**DOI:** 10.1038/s41467-020-18959-8

**Published:** 2020-10-09

**Authors:** Sun-Ting Tsai, En-Jui Kuo, Pratyush Tiwary

**Affiliations:** 1 Department of Physics and Institute for Physical Science and Technology, University of Maryland, College Park, MD 20742 USA; 2 Department of Physics and Joint Quantum Institute, University of Maryland, College Park, MD 20742 USA; 3 Department of Chemistry and Biochemistry and Institute for Physical Science and Technology, University of Maryland, College Park, MD 20742 USA

**Keywords:** Biological physics, Chemical physics, Condensed-matter physics, Information theory and computation, Statistical physics, thermodynamics and nonlinear dynamics

## Abstract

Recurrent neural networks have led to breakthroughs in natural language processing and speech recognition. Here we show that recurrent networks, specifically long short-term memory networks can also capture the temporal evolution of chemical/biophysical trajectories. Our character-level language model learns a probabilistic model of 1-dimensional stochastic trajectories generated from higher-dimensional dynamics. The model captures Boltzmann statistics and also reproduces kinetics across a spectrum of timescales. We demonstrate how training the long short-term memory network is equivalent to learning a path entropy, and that its embedding layer, instead of representing contextual meaning of characters, here exhibits a nontrivial connectivity between different metastable states in the underlying physical system. We demonstrate our model’s reliability through different benchmark systems and a force spectroscopy trajectory for multi-state riboswitch. We anticipate that our work represents a stepping stone in the understanding and use of recurrent neural networks for understanding the dynamics of complex stochastic molecular systems.

## Abstract (web summary)

Artificial neural networks have been successfully used for language recognition. Tsai et al. use the same techniques to link between language processing and prediction of molecular trajectories and show capability to predict complex thermodynamics and kinetics arising in chemical or biological physics.

## Introduction

Recurrent neural networks (RNN) are a machine learning/artificial intelligence (AI) technique developed for modeling temporal sequences, with demonstrated successes including but not limited to modeling human languages^1–7^. A specific and extremely popular instance of RNNs are long short-term memory (LSTM)^8^ neural networks, which possess more flexibility and can be used for challenging tasks such as language modeling, machine translation, and weather forecasting^6,9,10^. LSTMs were developed to alleviate the limitation of previously existing RNN architectures wherein they could not learn information originating from far past in time. This is known as the vanishing gradient problem, a term that captures how the gradient or force experienced by the RNN parameters vanishes as a function of how long ago did the change happen in the underlying data^11,12^. LSTMs deal with this problem by controlling flows of gradients through a so-called gating mechanism where the gates can open or close determined by their values learned for each input. The gradients can now be preserved for longer sequences by deliberately gating out some of the effects. This way it has been shown that LSTMs can accumulate information for a long period of time by allowing the network to dynamically learn to forget aspects of information. Very recently LSTMs have also been shown to have the potential to mimic trajectories produced by experiments or simulations^13^, making accurate predictions about a short time into the future, given access to a large amount of data in the past. Similarly, another RNN variant named reservoir computing^14^ has been recently applied to learn and predict chaotic systems^15^. Such a capability is already useful for instance in weather forecasting, where one needs extremely accurate predictions valid for a short period of time. In this work, we consider an alternate and arguably novel use of RNNs, specifically LSTMs, in making predictions that in contrast to previous work^13,15^, are valid for very long periods of time but only in a statistical sense. Unlike domains such as weather forecasting or speech recognition where LSTMs have allowed very accurate predictions albeit valid only for short duration of time, here we are interested in problems from chemical and biological physics, where the emphasis is more on making statistically valid predictions valid for extremely long duration of time. This is typified for example through the use of the ubiquitous notion of rate constant for activated barrier crossing, where short-time movements are typically treated as noise, and are not of interest for being captured through a dynamical model.

Here we suggest an alternative way to use LSTM-based language model to learn a probabilistic model from the time sequence along some low-dimensional order parameters produced by computer simulations or experiments of a high-dimensional system. We also show by our computer simulations of different model systems that the language model can produce the correct Boltzmann statistics (as can other AI methods such as refs. ^16,17^) but also the kinetics over a large spectrum of modes characterizing the dynamics in the underlying data. We highlight here a unique aspect of this calculation that the order parameter our framework needs could be arbitrarily far from the true underlying slow mode, often called reaction coordinate. This in turn dictates how long of a memory kernel must be captured which is in general a very hard problem to solve^18,19^. Our framework is agnostic to proximity from the true reaction coordinate and reconstructs statistically accurate dynamics in a wide range of order parameters. We also show how the minimization of loss function leads to learning the path entropy of a physical system, and establish a connection between the embedding layer and transition probability. Followed by this connection, we also show how we can define a transition probability through embedding vectors. We provide tests for Boltzmann statistics and kinetics for Langevin dynamics of model potentials, MD simulation of alanine dipeptide, and trajectory from single molecule force spectroscopy experiment on a multi-state riboswitch^20^, respectively. We also compare our protocol with alternate approaches including Hidden Markov Models. Our work thus represents a new usage of a popular AI framework to perform dynamical reconstruction in a domain of potentially high fundamental and practical relevance, including materials and drug design.

## Results

### Molecular dynamics can be mapped into a sequence of characters

**Fig. 1:**
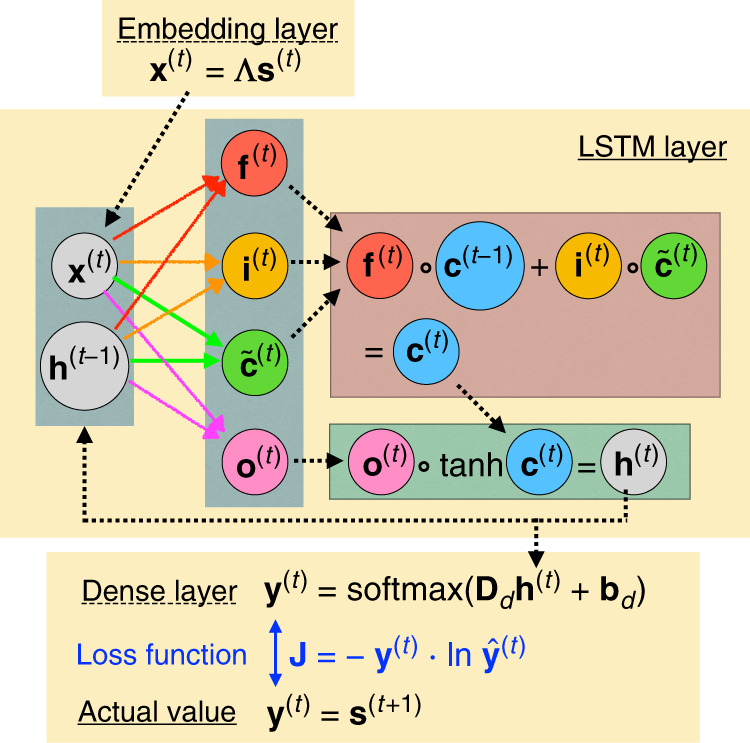
Neural network schematic. The schematic plot of the simple character-level language model used in this work. The model consists of three main parts: The embedding layer, the LSTM layer, and a dense output layer. The embedding layer is a linear layer which multiplies the one-hot input **s**^(*t*)^ by a matrix and produces an embedding vector **x**^(*t*)^. The **x**^(*t*)^ is then used as the input of LSTM network, in which the forget gate **f**^(*t*)^, the input gate **i**^(*t*)^, the output gate **o**^(*t*)^, and the candidate value ${\tilde{{\bf{c}}}}^{(t)}$ are all controlled by (**x**^(*t*)^, **h**^(*t*−1)^). The forget gate and input gate are then used to produce the update equation of cell state **c**^(*t*)^. The output gate decides how much information propagates to the next time step. The output layer predicts the probabilities ${\hat{{\bf{y}}}}^{(t)}$ by parametrizing the transformation from **h**^(*t*)^ to $\hat{{\bf{y}}}$ with learned weights **D**_*d*_ and learned biases **b**_*d*_. Finally, we can compute the cross entropy between the predicted probability distribution ${\hat{{\bf{y}}}}^{(t)}$ and the true probability distribution **y**^(*t*)^ = **s**^(*t*+1)^.

Our central rationale in this work is that molecular dynamics (MD) trajectories, adequately discretized in space and time, can be mapped into a sequence of characters in some languages. By using a character-level language model that is effective in predicting future characters given the characters so far in a sequence, we can learn the evolution of the MD trajectory that was mapped into the characters. The model we use is stochastic since it learns each character through the probability they appear in a corpus used for training. This language model consists of three sequential parts shown schematically in Fig. 1. First, there is an embedding layer mapping one-hot vectors to dense vectors, followed by an LSTM layer which connects input states and hidden states at different time steps through a trainable recursive function, and finally a dense layer to transform the output of LSTM to the categorical probability vector.

Specifically, here we consider as input a one-dimensional time series produced by a physical system, for instance through Langevin dynamics being undergone by a complex molecular system. The time series consist of data points {*ξ*^(*t*)^}, where *t* labels the time step and $\xi \in {\mathbb{R}}$ is some one-dimensional collective variable or order parameter for the high-dimensional molecular system. In line with standard practice for probabilistic models, we convert the data points to one-hot encoded representations that implement spatial discretization. Thus each data point {*ξ*^(*t*)^} is represented by a *N*-dimensional binary vector **s**^(*t*)^, where *N* is the number of discrete grid-points. An entry of one stands for the representative value and all the other entries are set to zeros. The representative values are in general finite if the order parameter is bounded, and are equally spaced in ${\mathbb{R}}$ with in total *N* representative values. Note that the time series {*ξ*^(*t*)^} does not have to be one-dimensional. For a higher-dimensional series, we can always choose a set of representative values corresponding to locations in the higher-dimensional space visited trajectory. This would typically lead to a larger *N* in the one-hot encoded representations, but the training set size itself will naturally stay the same. We find that the computational effort only depends on the size of training set and very weakly on *N*, and thus the time spent for learning a higher dimensional time series does not increase much relative to a one-dimensional series.

In the sense of modeling languages, the one-hot representation on its own cannot capture the relation between different characters. Take for instance that there is no word in the English language where the character *c* is followed by *x*, unless of course one allows for the possibility of a space or some other letter in between. To deal with this, computational linguists make use of an embedding layer. The embedding layer works as a look-up table which converts each one-hot vector **s**^(*t*)^ to a dense vector ${{\bf{x}}}^{(t)}\in {{\mathbb{R}}}^{M}$ by the multiplication of a matrix **Λ** which is called the embedding matrix, where *M* is called the embedding dimension

1 $${{\bf{x}}}^{(t)}={\Lambda }{{\bf{s}}}^{(t)}$$

The sequence of dense representation **x**^(*t*)^ accounts for the relation between different characters as seen in the training time series. **x**^(*t*)^ is then used as the input of the LSTM layer. Each **x**^(*t*)^ generates an output ${{\bf{h}}}^{(t)}\in {{\mathbb{R}}}^{L}$ from LSTM layer, where *L* is a tunable hyperparameter. Larger *L* generally gives better learning capability but needs more computational resources. The LSTM itself consists of the following elements: the input gate **i**^(*t*)^, the forget gate **f**^(*t*)^, the output gate **o**^(*t*)^ the cell state **c**^(*t*)^, the candidate value ${\tilde{{\bf{c}}}}^{(t)}$, and **h**^(*t*)^ which is the hidden state vector and the final output from the LSTM. Each gate processes information in different aspects.^8^ Briefly, the input gate decides which information to be written, the forget gate decides which information to be erased, and the output gate decides which information to be read from the cell state to the hidden state. The update equation of these elements can be written as follows:

2 $${{\bf{f}}}^{(t)}=\sigma ({{\bf{W}}}_{f}{{\bf{x}}}^{(t)}+{{\bf{U}}}_{f}{{\bf{h}}}^{(t-1)}+{{\bf{b}}}_{f})$$

3 $${{\bf{i}}}^{(t)}=\sigma ({{\bf{W}}}_{i}{{\bf{x}}}^{(t)}+{{\bf{U}}}_{i}{{\bf{h}}}^{(t-1)}+{{\bf{b}}}_{i})$$

4 $${{\bf{o}}}^{(t)}=\sigma ({{\bf{W}}}_{o}{{\bf{x}}}^{(t)}+{{\bf{U}}}_{o}{{\bf{h}}}^{(t-1)}+{{\bf{b}}}_{o})$$

5 $$\quad\; {\tilde{{\bf{c}}}}^{(t)}=\tanh ({{\bf{W}}}_{c}{{\bf{x}}}^{(t)}+{{\bf{U}}}_{c}{{\bf{h}}}^{(t-1)}+{{\bf{b}}}_{c})$$

6 $$\,{{\bf{c}}}^{(t)}={{\bf{f}}}^{(t)}\circ {{\bf{c}}}^{(t-1)}+{{\bf{i}}}^{(t)}\circ {\tilde{{\bf{c}}}}^{(t)}\qquad$$

7 $$\;{{\bf{h}}}^{(t)}={{\bf{o}}}^{(t)}\circ \tanh ({{\bf{c}}}^{(t)})\qquad\qquad\quad$$

where **W** and **b** are the corresponding weight matrices and bias vectors. The $\tanh ({\bf{v}})$ operates piecewise on each element of the vector **v**. The operation ∘ is the Hadamard product^21^.

The final layer in Fig. 1 is a simple dense layer with fully connected neurons which converts the output **h**^(*t*)^ of the LSTM to a vector **y**^(*t*)^ in which each entry denotes the categorical probability of the representative value for the next time step *t* + 1. The loss function *J* for minimization during training at every timestep *t* is then defined as the cross entropy between the output of the model ${\hat{{\bf{y}}}}^{(t)}$ and the actual probability for the next timestep ${{\bf{y}}}^{(t)}$ which is just the one-hot vector **s**^*t*+1^

8 $${\hat{{\bf{y}}}}^{(t)}={\rm{softmax}}({{\bf{D}}}_{d}{{\bf{h}}}^{(t)}+{{\bf{b}}}_{d})$$

9 $$J=-\sum_{t = 0}^{T-1}{{\bf{y}}}^{(t)}\cdot {\mathrm{ln}}\,{\hat{{\bf{y}}}}^{(t)}=-\sum_{t = 0}^{T-1}{{\bf{s}}}^{(t+1)}\cdot {\mathrm{ln}}\,{\hat{{\bf{y}}}}^{(t)}$$

where *T* is the total length of trajectory, and the final loss function is the sum over the whole time series. The ${\rm{softmax}}{({\bf{x}})}_{i}=\exp ({{\bf{x}}}_{i})/{\sum }_{j}\exp ({{\bf{x}}}_{j})$ is a softmax function mapping **x** to a probability vector $\hat{{\bf{y}}}$.

### Training the network is equivalent to learning path entropy

The central finding of this work, which we demonstrate through numerical results for different systems, is that a LSTM framework used to model languages can also be used to capture kinetic and thermodynamic aspects of dynamical trajectories prevalent in chemical and biological physics. In this section we demonstrate theoretically as to why LSTMs possess such a capability. Before we get into the mathematical reasoning detailed here, as well as in Supplementary Note 1, we first state our key idea. Minimizing the loss function *J* in LSTM (Eq. (9)), which trains the model at time *t* to generate output ${\hat{{\bf{y}}}}^{(t)}$ resembling the target output **s**^*t*+1^, is equivalent to minimizing the difference between the actual and LSTM-learned path probabilities. This difference between path probabilities can be calculated as a cross-entropy $J^{\prime}$ defined as:

10 $$J^{\prime} =-\sum_{{{\bf{x}}}^{(T)}...{{\bf{x}}}^{(0)}}P({{\bf{x}}}^{(T)}...{{\bf{x}}}^{(0)}){\mathrm{ln}}\,Q({{\bf{x}}}^{(T)}...{{\bf{x}}}^{(0)})$$

where *P*(**x**^(*t*+1)^, . . . , **x**^(0)^) and *Q*(**x**^(*t*+1)^, . . . , **x**^(0)^) are the corresponding true and neural network learned path probabilities of the system. Equation (10) can be rewritten^22^ as the sum of path entropy *H*(*P*) for the true distribution *P* and Kullback–Liebler distance *D*_*KL*_ between *P* and *Q*: $J^{\prime} =H(P)+{D}_{KL}(P| | Q)$. Since *D*_*KL*_ is strictly non-negative^22^ attaining the value of 0 iff *Q* = *P*, the global minimum of $J^{\prime}$ happens when *Q* = *P* and $J^{\prime}$ equals the path entropy *H*(*P*) of the system.^23^ Thus we claim that minimizing the loss function in LSTM is equivalent to learning the path entropy of the underlying physical model, which is what makes it capable of capturing kinetic information of the dynamical trajectory.

To prove this claim we start with rewriting *J* in Eq. (9). For a long enough observation period *T* or for a very large number of trajectories, *J* can be expressed as the cross entropy between conditional probabilities:

11 $$J=	 \, -\sum_{t = 0}^{T-1}\sum_{{{\bf{x}}}^{(t+1)}} P({{\bf{x}}}^{(t+1)}| {{\bf{x}}}^{(t)}...{{\bf{x}}}^{(0)})\\ 	\,\, \times {\mathrm{ln}}\,Q({{\bf{x}}}^{(t+1)}| {{\bf{x}}}^{(t)}...{{\bf{x}}}^{(0)})$$

where *P*(**x**^(*t*+1)^∣**x**^(*t*)^. . . **x**^(0)^) is the true conditional probability for the physical system, and *Q*(**x**^(*t*+1)^∣**x**^(*t*)^. . . **x**^(0)^) is the conditional probability learned by the neural network. The minimization of Eq. (11) leads to minimization of the cross entropy $J^{\prime}$ as shown in the SI. Here we conversely show how Eq. (10) reduces to Eq. (9) by assuming a stationary first-order Markov process as in ref. ^23^:

12 $$\begin{array}{l}P({{\bf{x}}}^{(T)}...{{\bf{x}}}^{(0)})=P({{\bf{x}}}^{(T)}| {{\bf{x}}}^{(T-1)})...P({{\bf{x}}}^{(1)}| {{\bf{x}}}^{(0)})P({{\bf{x}}}^{(0)})\\ Q({{\bf{x}}}^{(T)}...{{\bf{x}}}^{(0)})=Q({{\bf{x}}}^{(T)}| {{\bf{x}}}^{(T-1)})...Q({{\bf{x}}}^{(1)}| {{\bf{x}}}^{(0)})Q({{\bf{x}}}^{(0)})\end{array}$$

where $P({{\bf{x}}}_{j}^{(t+1)}| {{\bf{x}}}_{i}^{(t)})\equiv {P}_{ij}$ is the transition probability from state **x**_*i*_ to state **x**_*j*_ and $P({{\bf{x}}}_{k}^{(0)})\equiv {P}_{k}$ is the occupation probability for the single state **x**_*k*_. Plugging Eq. (12) into Eq. (10), and following the derivation in ref. ^23^ with the constraints

13 $$\sum_{j}{P}_{ij}=1\quad \sum_{i}{P}_{i}{P}_{ij}={P}_{j}$$

we arrive at an expression for the cross-entropy *J*, which is very similar to the path entropy type expressions derived for instance in the framework of Maximum Caliber^23^:

14 $$J^{\prime} =-\sum_{i}{P}_{i}{\mathrm{ln}}\,{Q}_{i}-T\sum_{lm}{P}_{l}{P}_{lm}{\mathrm{ln}}\,({Q}_{lm})$$

15 $$\to -T\sum_{lm}P({{\bf{x}}}_{l})P({{\bf{x}}}_{m}| {{\bf{x}}}_{l}){\mathrm{ln}}\,Q({{\bf{x}}}_{m}| {{\bf{x}}}_{l})$$

In Eq. (14) as the trajectory length *T* increases, the second term dominates in the estimate of *J* leading to Eq. (15). This second term is the ensemble average of a time-dependent quantity $\tilde{J}({{\bf{x}}}_{l}^{(t)})\equiv -{\sum }_{m}P({{\bf{x}}}_{m}^{(t+1)}| {{\bf{x}}}_{l}^{(t)}){\mathrm{ln}}\,Q({{\bf{x}}}_{m}^{(t+1)}| {{\bf{x}}}_{l}^{(t)})$. For a large enough *T*, the ensemble average can be replaced by the time average. By assuming ergodicity^24^:

16 $$J^{\prime} =-\sum_{t = 1}^{T}\sum_{m}P({{\bf{x}}}_{m}^{(t+1)}| {{\bf{x}}}_{l}^{(t)}){\mathrm{ln}}\,Q({{\bf{x}}}_{m}^{(t+1)}| {{\bf{x}}}_{l}^{(t)})$$

from which we directly obtain Eq. (9). Therefore, under first-order Markovianity and ergodicity, minimizing the loss function *J* of Eq. (9) is equivalent to minimizing $J^{\prime}$ and thereby learning the path entropy. In the SI we provide a proof for this statement that lifts the Markovianity assumption as well—the central idea there is similar to what we showed here.

### Embedding layer captures kinetic distances

In word embedding theory, the embedding layer provides a measure of similarity between words. However, from the path probability representation, it is unclear how the embedding layer works since the derivation can be done without embedding vectors **x**. To have an understanding to *Q*_*lm*_ in the first-order Markov process, we first write the conditional probability ${Q}_{lm}=Q({{\bf{x}}}_{m}^{(t+1)}| {{\bf{x}}}_{l}^{(t)})$ explicitly with softmax defined in Eq. (8) and embedding vectors **x** defined in Eq. (1):

17 $${Q}_{lm}	= \, \frac{\exp ({{\bf{s}}}_{m}^{(t+1)}\cdot ({{\bf{D}}}_{d}{{\bf{h}}}^{(t)}+{{\bf{b}}}_{d}))}{{\sum }_{k}\exp ({{\bf{s}}}_{k}\cdot ({{\bf{D}}}_{d}{{\bf{h}}}^{(t)}+{{\bf{b}}}_{d}))}\\ 	= \,\frac{\exp ({{\bf{s}}}_{m}^{(t+1)}\cdot ({{\bf{D}}}_{d}{f}_{{\boldsymbol{\theta }}}({{\bf{x}}}^{(t)})+{{\bf{b}}}_{d}))}{{\sum }_{k}\exp ({{\bf{s}}}_{k}\cdot ({{\bf{D}}}_{d}{f}_{{\boldsymbol{\theta }}}({{\bf{x}}}^{(t)})+{{\bf{b}}}_{d}))}$$

where *f* is the recursive function **h**^(*t*)^ = *f*_***θ***_(**x**^(*t*)^, **h**^(*t*−1)^) ≈ *f*_***θ***_(**x**^(*t*)^) which is defined with the update equation in Eq. (2)–(7). In Eq. (17), ***θ*** denotes various parameters including all weight matrices and biases, and the summation index *k* runs over all possible states. Now we can use multivariable Taylor’s theorem to approximate *f*_***θ***_ as the linear term around a point **a** as long as **a** is not at any local minimum of *f*_***θ***_:

18 $${f}_{{\boldsymbol{\theta }}}({{\bf{x}}}^{(t)})\approx {f}_{{\boldsymbol{\theta }}}({\bf{a}})+{{\bf{A}}}_{{\boldsymbol{\theta }}}({{\bf{x}}}^{(t)}-{\bf{a}})$$

where **A**_***θ***_ is the *L* by *M* matrix defined to be ${({{\bf{A}}}_{{\boldsymbol{\theta }}})}_{ij}=\frac{\partial {({f}_{{\boldsymbol{\theta }}})}_{i}}{\partial {x}_{j}}{| }_{{\bf{x}} = {\bf{a}}}$. Then Eq. (17) becomes

19 $${Q}_{lm}=\frac{\exp ({C}_{m}^{(t+1)})\exp ({{\bf{s}}}_{m}^{(t+1)}\cdot {{\bf{D}}}_{d}{{\bf{A}}}_{{\boldsymbol{\theta }}}{{\bf{x}}}_{l}^{(t)})}{{\sum }_{k}\exp ({C}_{k})\exp ({{\bf{s}}}_{k}\cdot {{\bf{D}}}_{d}{{\bf{A}}}_{{\boldsymbol{\theta }}}{{\bf{x}}}_{l}^{(t)})}$$

where ${C}_{i}^{(t+1)}={{\bf{s}}}_{i}^{(t+1)}\cdot [{{\bf{D}}}_{d}({f}_{{\boldsymbol{\theta }}}({{\bf{a}}}_{l})+{{\bf{A}}}_{{\boldsymbol{\theta }}}{{\bf{a}}}_{l})+{{\bf{b}}}_{d}]$. We can see in Eq. (19) how the embedding vectors come into the transition probability. Specifically, there is a symmetric form between output one-hot vectors ${{\bf{s}}}_{m}^{(t+1)}$ and the input one-hot vectors **s**^(*t*)^, in which **x**^(*t*)^ = **Λs**^(*t*)^ and **Λ** is the input embedding matrix, **D**_*d*_**A**_***θ***_ can be seen as the output embedding matrix, and ${C}_{i}^{(t+1)}$ is the correction of time lag effect. While we do not have an explicit way to calculate the output embedding matrix so defined, Eq. (19) motivates us to define the following ansatz for the transition probability:

20 $${Q}_{lm}=Q({{\bf{x}}}_{m}| {{\bf{x}}}_{l})=\frac{\exp ({{\bf{x}}}_{m}\cdot {{\bf{x}}}_{l})}{{\sum }_{k}\exp ({{\bf{x}}}_{k}\cdot {{\bf{x}}}_{l})}$$

where **x**_*m*_ and **x**_*l*_ are both calculated by the input embedding matrix **Λ**. The expression in Eq. (20) is thus a tractable approximation to the more exact transition probability in Eq. (19). Furthermore, we show through numerical examples of test systems that our ansatz for *Q*_*lm*_ does correspond to the kinetic connectivity between states. That is, the LSTM embedding layer with the transition probability through Eq. (20) can capture the average commute time between two states in the original physical system, irrespective of the quality of low-dimensional projection fed to the LSTM^25–27^.

### Test systems

To demonstrate our ideas, here we consider a range of different dynamical trajectories. These include three model potentials, the popular model molecule alanine dipeptide, and trajectory from single molecule force spectroscopy experiments on a multi-state riboswitch.^20^ The sample trajectories of these test systems and the data preprocessing strategies are put in the Supplementary Note 5 and Supplementary Figs. 14–18 When applying our neural network to the model systems, the embedding dimension *M* is set to 8 and LSTM unit *L* set to 64. When learning trajectories for alanine dipeptide and riboswitch, we took *M* = 128 and *L* = 1024. All time series were batched into sequences with a sequence length of 100 and the batch size of 64. For each model potential, the neural network was trained using the method of stochastic gradient descent for 20 epochs until the training loss becomes smaller than the validation loss, which means an appropriate training has been reached. For alanine dipeptide, 40 training epochs were used. Our neural network was built using TensorFlow version 1.10. Further system details are provided in “Methods” section.

### Boltzmann statistics and kinetics for model potentials

**Fig. 2:**
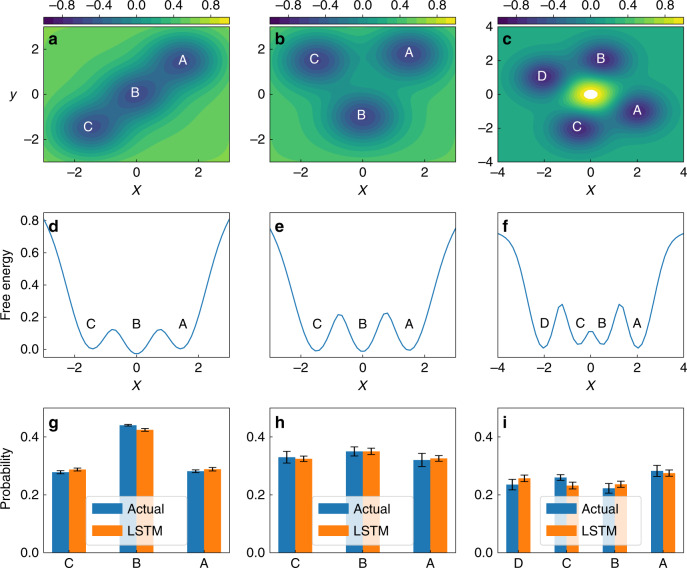
Boltzmann statistics for model systems. The analytical free energy generated from **a** linear 3-state, **b** triangular 3-state, **c** symmetric 4-state model potentials and **d**, **e**, **f** are the corresponding 1-dimensional projections along *x*-direction. In the bottom, we compare the Boltzmann probabilities of **g** linear 3-state, **h** triangular 3-state, and **i** symmetric 4-state models for every labeled state generated from actual MD simulation and from our long short-term memory (LSTM) network. The errorbars are calculated as standard errors.

The first test we perform for our LSTM set-up is its ability to capture the Boltzmann weighted statistics for the different states in each model potential. This is the probability distribution *P* or equivalently the related free energy $F=-\frac{1}{\beta }\,\text{log}\,P$, and can be calculated by direct counting from the trajectory. As can be seen in Fig. 2, the LSTM does an excellent job of recovering the Boltzmann probability within error bars.

Next we describe our LSTM deals with a well-known problem in analyzing high-dimensional data sets through low-dimensional projections. One can project the high-dimensional data along many different possible low-dimensional order parameters, for instance *x*, *y,* or a combination thereof in Fig. 2. However most such projections will end up not being kinetically truthful and give a wrong impression of how distant the metastable states actually are from each other in the underlying high-dimensional space. It is in general hard to come up with a projection that preserves the kinetic properties of the high-dimensional space. Consequently, it is hard to design analysis or sampling methods that even when giving a time-series along a sub-optimal projection, still capture the true kinetic distance in the underlying high-dimensional space.

Here we show how our LSTM model is agnostic to the quality of the low-dimensional projection in capturing accurate kinetics. Given that for each of the 3 potentials the LSTM was provided only the *x*−trajectory, we can expect that the chosen model potentials constitute different levels of difficulties in generating correct kinetics. Specifically, a one-dimensional projection along *x* is kinetically truthful for the linear 3-state potential in Fig. 2a but not for the triangular 3-state and the 4-state potentials in Fig. 2b and c, respectively. For instance, Fig. 2e gives the impression that state C is kinetically very distant from state A, while in reality for this potential all 3 pairs of states are equally close to each other. Similar concerns apply to the 4-state potential.

**Fig. 3:**
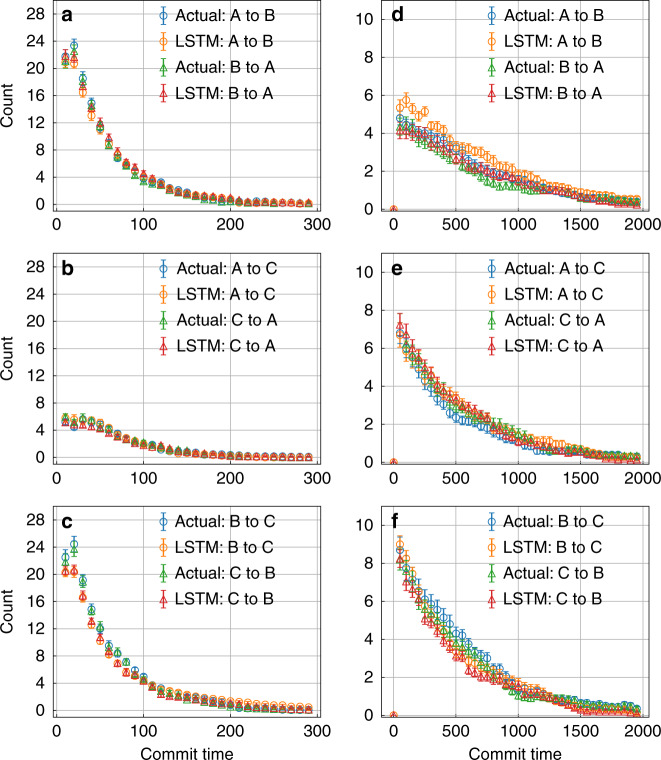
Kinetics for 3-state model systems. Number of transitions between different pairs of metastable states as a function of commitment time defined in “Results” section. The calculations for linear and triangular configurations are shown in **a**–**c** and **d**–**f**, respectively. Error bars are illustrated and were calculated as standard errors.

**Fig. 4:**
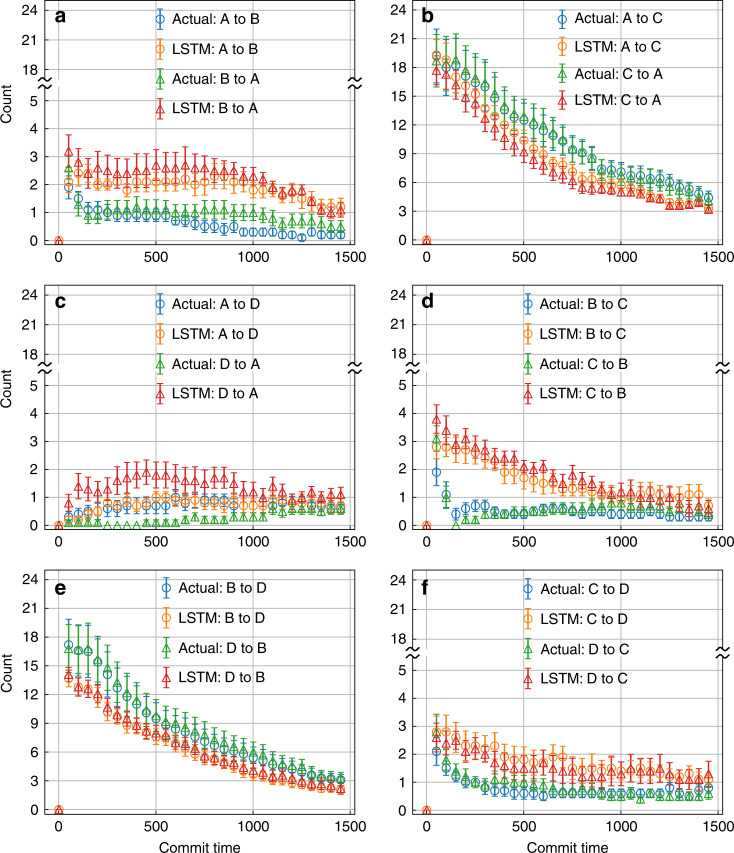
Kinetics for 4-state model system. Number of transitions between different pairs of metastable states as a function of commitment time defined in “Results” section for 4-state model system. Error bars are illustrated and were calculated as standard errors.

In Figs. 3 and 4a–c and d–f we compare the actual versus LSTM-predicted kinetics for moving between different metastable states for different model potentials, for all pairs of transitions in both directions (i.e., for instance A to B and B to A). Specifically, Fig. 3a–c and 3d–f shows results for moving between the 3 pairs of states in the linear and triangular 3-state potentials, respectively. Figure 4 shows results for the 6 pairs of states in the 4-state potential. Furthermore, for every pair of state, we analyze the transition time between those states as a function of different minimum commitment or commit time, i.e., the minimum time that must be spent by the trajectory in a given state to be classified as having committed to it. A limiting value, and more specifically the rate at which the population decays to attain to such a limiting value, corresponds to the inverse of the rate constant for moving between those states^28,29^. Thus here we show how our LSTM captures not just the rate constant, but time-dependent fluctuations in the population in a given metastable state as equilibrium is attained. The results are averaged over 20 independent segments taken from the trajectories of different trials of training for the 3-state potentials and 10 independent segments for the 4-state potential.

As can be seen in Figs. 3 and 4, the LSTM model does an excellent job of reproducing well within errorbars the transition times between different metastable states for different model potentials irrespective of the quality of the low-dimensional projection. Firstly, our model does tell the differences between linear and triangular 3-state models (Fig. 3) even though the projected free energies along the *x* variable input into LSTM are same (Fig. 2). The number of transitions between states A and C is less than the others; while for triangular configuration, the numbers of transitions between all pairs of states are similar. The rates at which the transition count decays as a function of commitment time is also preserved between the input data and the LSTM prediction.

The next part of our second test is the 4-state model potential. In Fig. 4 we show comparisons for all 6 pairs of transitions in both forward and reverse directions. A few features are immediately striking here. Firstly, even though states B and C are perceived to be kinetically proximal from the free energy (Fig. 2), the LSTM captures that they are distal from each other and correctly assigns similar kinetic distance to the pairs B, C as it does to A, D. Secondly, there is asymmetry between the forward and backward directions (for e.g., A to D and D to A, indicating that the input trajectory itself has not yet sufficiently sampled the slow transitions in this potential. As can be seen from Fig. 2c the input trajectory has barely 1 or 2 direct transitions for the very high barrier A to D or B to C. This is a likely explanation for why our LSTM model does a bit worse than in the other two model potentials in capturing the slowest transition rates, as well as the higher error bars we see here. In other words, so far we can conclude that while our LSTM model can capture equilibrium probabilities and transition rates for different model potentials irrespective of the input projection direction or order parameter, it is still not a panacea for insufficient sampling itself, as one would expect.

### Boltzmann statistics and kinetics for alanine dipeptide

**Fig. 5:**
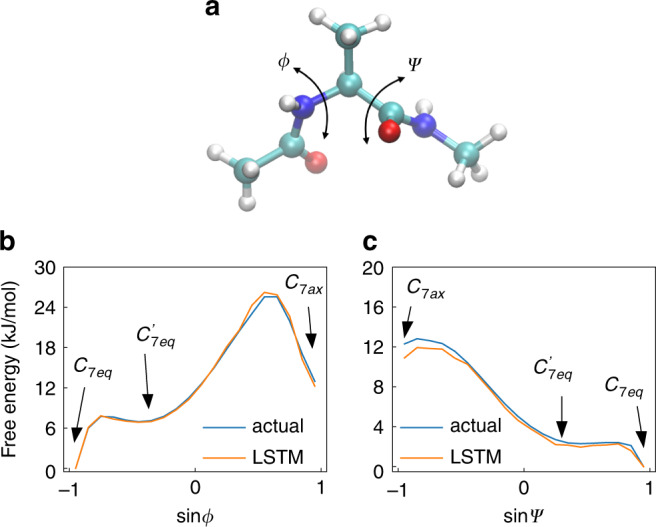
Boltzmann statistics for alanine dipeptide. **a** The molecular structure of alanine dipeptide used in the actual MD simulation. The torsional angles *ϕ* and *ψ* as the collective variables (CVs) are shown. **b** and **c** The 1-dimensional free energy curves along $\sin \phi$ and $\sin \psi$ are calculated using actual MD data and the data generated from LSTM. For the calculation of a different epoch, please see Supplementary Note 2 and Supplementary Table 1.

Finally, we apply our LSTM model to the study of conformational transitions in alanine dipeptide, a model biomolecular system comprising 22 atoms, experiencing thermal fluctuations when coupled to a heat bath. The structure of alanine dipeptide is shown in Fig. 5a. While the full system comprises around 63 degrees of freedom, typically the torsional angles *ϕ* and *ψ* are used to identify the conformations of this peptide. Over the years a large number of methods have been tested on this system in order to perform enhanced sampling of these torsions, as well as to construct optimal reaction coordinates^30–33^. Here we show that our LSTM model can very accurately capture the correct Boltzmann statistics, as well as transition rates for moving between the two dominant metastable states known as *C*_7*eq*_ and *C*_7*ax*_. Importantly, the reconstruction of the equilibrium probability and transition kinetics, as shown in Fig. 5 and Table 1 is extremely accurate irrespective of the choice of one-dimensional projection time series fed into the LSTM. Specifically, we do this along $\sin \phi$ and $\sin \psi$, both of which are known to quite distant from an optimized kinetically truthful reaction coordinate^19,34^, where again we have excellent agreement between input and LSTM-predicted results.

**Table 1:** Kinetics for alanine dipeptide.

Alanine dipeptide			
CVs	Label	*C*_7*eq*_ to *C*_7*ax*_ (*ps*)	*C*_7*ax*_ to *C*_7*eq*_ (*ps*)
$\sin \phi$	Actual	5689.22 ± 962.366	107.93 ± 11.267
	LSTM	5752.16 ± 710.399	103.81 ± 14.268
$\sin \psi$	Actual	5001.42 ± 643.943	105.70 ± 13.521
	LSTM	4325.01 ± 526.293	81.68 ± 10.288

Inverse of transition rates for conformational transitions in alanine dipetide calculated from actual MD trajectories of LSTM model. Here we show the calculation along two different CVs: $\sin \phi$ and $\sin \psi$.

### Learning from single molecule force spectroscopy trajectory

**Fig. 6:**
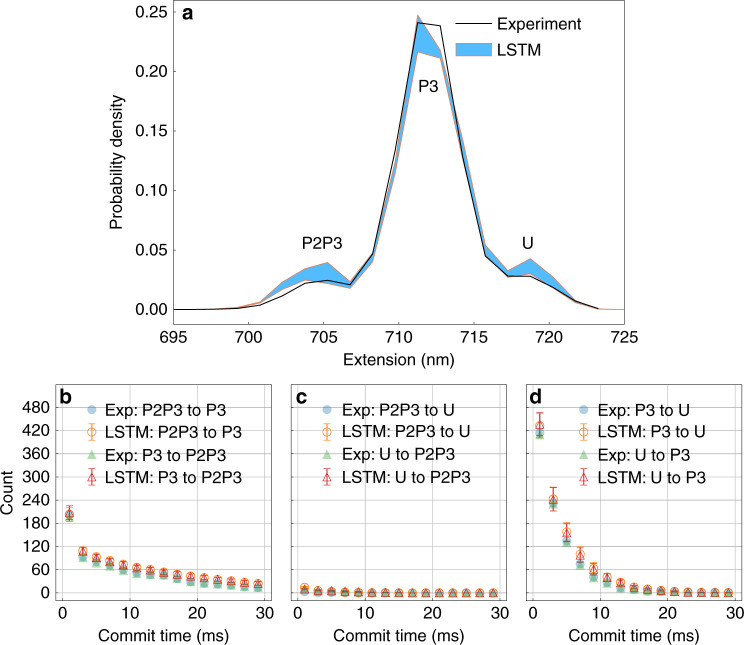
Boltzmann statistics and kinetics for riboswitch. Using LSTM model to learn thermodynamics and kinetics from a folding and unfolding trajectory taken from a single molecule force spectroscopy measurement^20^: **a** Comparison between the probability density learned by the LSTM model and calculated from the experimental data. The regions between errorbars defined as standard errors are filled with blue color. **b**–**d** Commit time plots calculated by counting the transitions in the trajectory generated by LSTM and the experimental trajectory. The commit time is the minimum time that must be spent by the trajectory in a given state to be classified as having committed to it. Error bars are illustrated and were calculated as standard errors.

In this section, we use our LSTM model to learn from single molecule force spectroscopy experiments of a multi-state riboswitch performed with a constant force of 10.9 pN. The data points are measured at 10 kHz (i.e., every 100 μs). Other details of the experiments can be found in ref. ^20^. The trajectory for a wide range of extensions starting 685 nm up to 735 nm was first spatially discretized into 34 labels, and then converted to a time series of one hot vectors, before being fed into the LSTM model. The results are shown in Fig. 6. In Fig. 6a, we have shown an agreement between a profile of probability density averaged over 5 independent training sets with the probability density calculated from the experimental data. Starting from the highest extension, the states are fully unfolded (U), longer intermediate (P3) and shorter intermediate (P2P3)^20^. From Fig. 6b–c, we see that the LSTM model captures the kinetics for moving between all 3 pairs of states for a very wide range of commitment times.

### Embedding layer based kinetic distance

In Eq. (19), we derived a non-tractable relation for conditional transition probability in the embedding layer, and then through Eq. (20) we introduced a tractable ansatz in the spirit of Eq. (19). Here we revisit and numerically validate Eq. (20). Specifically, given any two embedding vectors **x**_*l*_ and **x**_*m*_ calculated from any two states *l* and *m*, we estimate the conditional probability *Q*_*lm*_ using Eq. (20). We use *Q*_*i*_ to denotes the Boltzmann probability predicted by the LSTM model. We then write down the interconversion probability *k*_*lm*_ between states *l* and *m* as:

21 $$\begin{array}{r}{k}_{lm}={Q}_{l}{Q}_{lm}+{Q}_{m}{Q}_{ml}\equiv 1/{t}_{lm}\end{array}$$

**Fig. 7:**
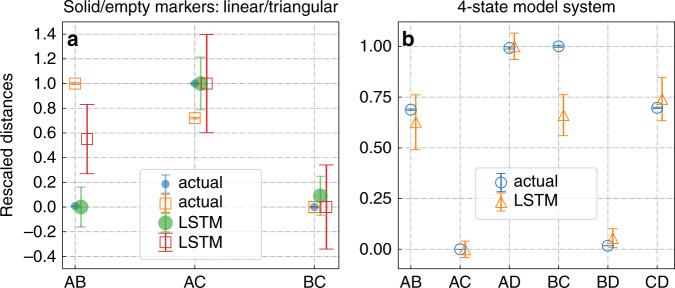
Analysis of embedding layers for model systems. Our analysis of the embedding layer constructed for **a** the linear and triangular 3-state and **b** the 4-state model systems. In **a**, we use solid circle and empty square markers, respectively to represent linear and triangular 3-state model potentials. In each plot, the data points are shifted slightly to the right for clarity. The distances marked actual and LSTM represent rescaled mean transition times as per Eqs. (22) and (21), respectively. Error bars were calculated as standard errors over 50 different trajectories.

From inverting this rate we then calculate an LSTM-kinetic time as *t*_*lm*_ ≡ 1/*k*_*lm*_ = 1/(*Q*_*l*_*Q*_*lm*_ + *Q*_*m*_*Q*_*ml*_). In Fig. 7, we compare *t*_*lm*_ with the actual transition time *τ*_*lm*_ obtained from the input data, defined as

22 $${\tau }_{lm}=T/\langle {N}_{lm}\rangle$$

Here *N*_*lm*_ is the mean number of transitions between state *l* and *m*. As this number varies with the precise value of commitment time, we average *N*_*lm*_ over all commit times to get 〈*N*_*lm*_〉. These two timescales *t*_*lm*_ and *τ*_*lm*_ thus represent the average commute time or kinetic distance^25,26^ between two states *l* and *m*. To facilitate the comparison between these two very differently derived timescales or kinetic distances, we rescale and shift them to lie between 0 and 1. The results in Fig. 7 show that the embedding vectors display the connectivity corresponding to the original high-dimensional configuration space rather than those corresponding to the one-dimensional projection. The model captures the correct connectivity by learning kinetics, which is clear evidence that it is able to bypass the projection error along any degree of freedom. The result also explains how is it that no matter what degree of freedom we use, our LSTM model still gives correct transition times. As long as the degree of freedom we choose to train the model can be used to discern all metastable states, we can even use Eq. (20) to see the underlying connectivity. Therefore, the embedding vectors in LSTM can define a useful distance metric which can be used to understand and model dynamics, and are possibly part of the reason why LSTMs can model kinetics accurately inspite of quality of projection and associated non-Markvoian effects.

### Comparing with Markov state model and Hidden Markov Model

**Fig. 8:**
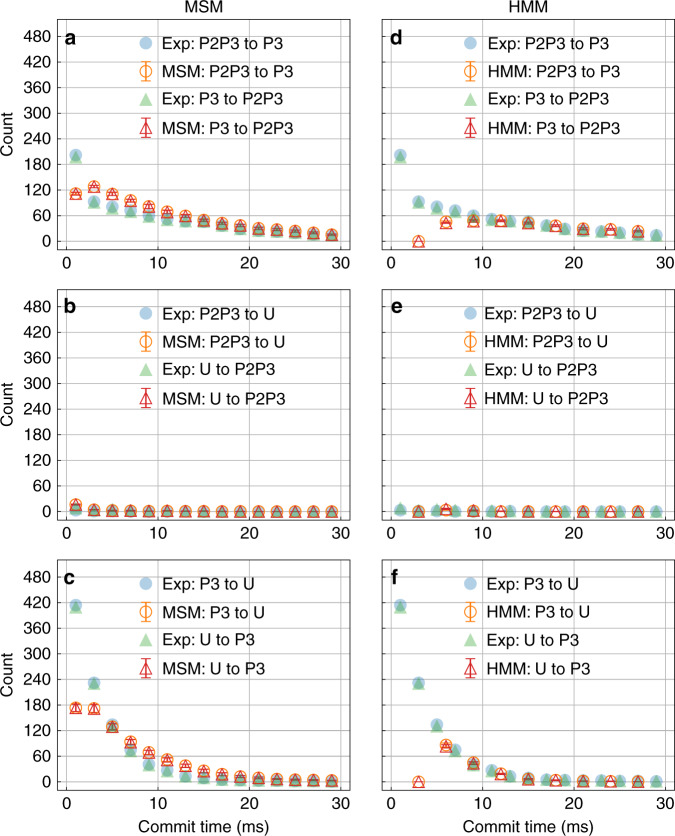
Riboswitch kinetics through alternate approaches. Number of transitions between different pairs of metastable states as a function of commitment time defined in “Results” section for the single molecule spectroscopy trajectory as learned by MSM (left column) and HMM (right column). Associated error bars calculated as standard errors are also provided.

In this section, we briefly compare our LSTM model with standard approaches for building kinetic models from trajectories, namely the Markov state model (MSM)^35^ and Hidden Markov model (HMM)^36–38^. Compared to LSTM, the MSM and HMM have smaller number of parameters, making them faster and more stable for simpler systems. However, both MSM and HMM require choosing an appropriate number of states and lag time^35,38,39^. Large number of pre-selected states or small lag time can lead to non-Markovian behavior and result in an incorrect prediction. Even more critically, choosing a large lag time also sacrifices the temporal precision. On the other hand, there is no need to determine the lag time and number of states using the LSTM network because LSTM does not rely on the Markov property. Choosing hyperparameters such as *M* and *L* may be comparable to choosing number of hidden states for HMM, while very similar values of *M* and *L* worked for systems as different as MD trajectory of alanine dipeptide and single molecule force spectroscopy trajectory of a riboswitch. At the same time, LSTM always generates the data points with the same temporal precision as it has in the training data irrespective of the intrinsic timescales it learns from the system. In Fig. 8, we provide the results of using HMM and MSM for the riboswitch trajectory with the same binning method and one-hot encoded input, to be contrasted with similar plots using LSTM in Fig. 6. Indeed both MSM and HMM achieve decent agreement with the true kinetics only if the commit time is increased approximately beyond 10 ms, while LSTM as shown in Fig. 6 achieved perfect agreement for all commit times. From this figure, it can be seen that the LSTM model achieves an expected agreement with as fine of a temporal precision as desired, even though we use 20 labels for alanine dipeptide and 34 labels for experimental data to represent the states. The computational efforts needed for the various approaches (LSTM, MSM, and HMM) are also provided in the Supplementary Note 3 and Supplementary Table 2–3, where it can be seen that LSTM takes similar amount of effort as HMM. The package we used to build the MSM and HMM is PyEMMA with version 2.5.6^40^. The models were built with lag time = 0.5 ms for MSM and lag time = 3 ms for HMM, where the HMM were built with number of hidden states = 3. A more careful comparison of the results along with analyses with other parameter choices such as different number of hidden states for HMM are provided in the Supplementary Note 4 and Supplementary Figs. 1–13, where we find all of these trends to persist.

## Discussion

In summary we believe this work demonstrates potential for using AI approaches developed for natural language processing such as speech recognition and machine translation, in unrelated domains such as chemical and biological physics. This work represents a first step in this direction, wherein we used AI, specifically LSTM flavor of recurrent neural networks, to perform kinetic reconstruction tasks that other methods^41,42^ could have also performed. We would like to argue that demonstrating the ability of AI approaches to perform tasks that one could have done otherwise is a crucial first step. In future works we will exploring different directions in which the AI protocol developed here could be used to perform tasks which were increasingly non-trivial in non-AI setups. More specifically, in this work we have shown that a simple character-level language model based on LSTM neural network can learn a probabilistic model of a time series generated from a physical system such as an evolution of Langevin dynamics or MD simulation of complex molecular models. We show that the probabilistic model can not only learn the Boltzmann statistics but also capture a large spectrum of kinetics. The embedding layer which is designed for encoding the contextual meaning of words and characters displays a nontrivial connectivity and has been shown to correlate with the kinetic map defined for reversible Markov chains^25,26,43^. An interesting future line of work for the embedding layer can be to uncover different states when they are incorrectly represented by the same reaction coordinate value, which is similar to finding different contextual meaning of the same word or character. For different model systems considered here, we could obtain correct timescales and rate constants irrespective of the quality of order parameter fed into the LSTM. As a result, we believe this kind of model outperforms traditional approaches for learning thermodynamics and kinetics, which can often be very sensitive to the choice of projection. Finally, the embedding layer can be used to define a new type of distance metric for high-dimensional data when one has access to only some low-dimensional projection. We hope that this work represents a first step in the use of RNNs for modeling, understanding and predicting the dynamics of complex stochastic systems found in biology, chemistry and physics.

## Methods

### Model potential details

All model potentials have two degrees of freedom *x* and *y*. Our first two models (shown in Fig. 2a and b) have three metastable states with governing potential *U*(*x*, *y*) given by

23 $$U(x,y)= 	\, W({x}^{6}+{y}^{6})-G(x,{x}_{1})G(y,{y}_{1})\\ \,	-G(x,{x}_{2})G(y,{y}_{2})-G(x,{x}_{3})G(y,{y}_{3})$$

where *W* = 0.0001 and $G(x,{x}_{0})={e}^{-\frac{{(x-x_0)}^{2}}{2{\sigma }^{2}}}$ denotes a Gaussian function centered at *x*_0_ with width *σ* = 0.8. We also build a 4-state model system with governing interaction potential:

24 $$U(x,y)= \,	 W({x}^{4}+{y}^{4})+G(x,0.0)G(y,0.0)\\ \, 	-G(x,2.0)G(y,-1.0)-G(x,0.5)G(y,2.0)\\ \,	- G(x,-0.5)G(y,-2.0)-G(x,-2.0)G(y,1.0)$$

The different local minima corresponding to the model potentials in Eq. (23) and Eq. (24) are illustrated in Fig. 2. We call these as linear 3-state, triangular 3-state, and 4-state models, respectively. The free energy surfaces generated from the simulation of Langevin dynamics^44^ with these model potentials are shown in Fig. 2a–c.

### Molecular dynamics details

The integration timestep for the Langevin dynamics simulation was 0.01 units, and the simulation was performed at *β* = 9.5 for linear 3-state and 4-state potentials and *β* = 9.0 for triangular 3-state potential, where *β* = 1/*k*_*B*_*T*. The MD trajectory for alanine dipeptide was obtained using the software GROMACS 5.0.4^45,46^, patched with PLUMED 2.4^47^. The temperature was kept constant at 450 K using the velocity rescaling thermostat^48^.

## Supplementary information

Supplementary Information

Peer Review File

## Data Availability

The single-molecule force spectroscopy experiment data for riboswitch was obtained from the authors of ref. ^20^ and they can be contacted for the same. All the other data associated with this work is available from the corresponding author on request.

## Code availability

MSM and HMM analyses were conducted with PyEMMA version 2.5.6.^40^ and available at http://www.pyemma.org. A Python based code of the LSTM language model is implemented using keras (https://keras.io/) with tensorflow-gpu (https://www.tensorflow.org/) as a backend, and available for public use at https://github.com/tiwarylab/LSTM-predict-MD.

## References

[1] Rico-Martinez R, Krischer K, Kevrekidis I, Kube M, Hudson J (1992). Discrete-vs. continuous-time nonlinear signal processing of cu electrodissolution data. Chem. Engg. Commun..

[2] Gicquel N, Anderson J, Kevrekidis I (1998). Noninvertibility and resonance in discrete-time neural networks for time-series processing. Phys. Lett. A.

[3] Graves A, Liwicki M, Fernández S, Bertolami R, Bunke H (2008). A novel connectionist system for unconstrained handwriting recognition. IEEE Trans. Pattern. Anal. Mach. Intell..

[4] Graves, A., Mohamed, A.-r. & Hinton, G. Speech recognition with deep recurrent neural networks. In *International Conference on Acoustics, Speech, and Signal Processing.* 6645–6649 (2013).

[5] Cho, K., Van Merriënboer, B., Gulcehre, C., Bougares, F., Schwenk, H., Bahdanau, D. & Bengio, Y. Learning phrase representations using rnn encoder-decoder for statistical machine translation. In *Proceedings of the 2014 Conference on Empirical Methods in Natural Language Processing (EMNLP)*. 1724–1734 (2014).

[6] Xingjian, S., Chen, Z., Wang, H. & Woo, W.-c. Convolutional lstm network: a machine learning approach for precipitation nowcasting. In *Advances in Neural Information Processing Systems*. 802–810 (2015).

[7] Chen, K., Zhou, Y. & Dai, F. A LSTM-based method for stock returns prediction: a case study of china stock market. In *IEEE**International Conference on Big Data*. 2823–2824 (2015).

[8] Hochreiter S, Schmidhuber J (1997). Long short-term memory. Neur. Comp..

[9] Sundermeyer, M., Schlüter, R. & Ney, H. LSTM neural networks for language modeling. In *Thirteenth Annual Conference of the International Speech Communication Association*. (2012).

[10] Luong, M.-T., Sutskever, I., Le, Q. V., Vinyals, O. & Zaremba, W. Addressing the rare word problem in neural machine translation. In *Proceedings of the 53rd Annual Meeting of the Association for Computational Linguistics and the 7th International Joint Conference on Natural Language Processing.* 11–19 (2014).

[11] Hochreiter, S. et al. Gradient flow in recurrent nets: the difficulty of learning long-term dependencies. (2001).

[12] Agar JC, Naul B, Pandya S, van Der Walt S (2019). Revealing ferroelectric switching character using deep recurrent neural networks. Nat. Commun..

[13] Eslamibidgoli, M. J., Mokhtari, M. & Eikerling, M. H. Recurrent neural network-based model for accelerated trajectory analysis in aimd simulations. Preprint at https://arxiv.org/abs/1909.10124 (2019).

[14] Lukoševičius M, Jaeger H (2009). Reservoir computing approaches to recurrent neural network training. Comp. Sci. Rev..

[15] Pathak J, Hunt B, Girvan M, Lu Z, Ott E (2018). Model-free prediction of large spatiotemporally chaotic systems from data: A reservoir computing approach. Phys. Rev. Lett..

[16] Noé F, Olsson S, Köhler J, Wu H (2019). Boltzmann generators: sampling equilibrium states of many-body systems with deep learning. Science.

[17] Sidky H, Chen W, Ferguson A (2020). Molecular latent space simulators. Chem. Sci.

[18] Bussi G, Laio A, Köhler J, Wu H (2020). Using metadynamics to explore complex free-energy landscapes. Nat. Rev. Phys..

[19] Wang Y, Ribeiro JML, Tiwary P (2019). Past–future information bottleneck for sampling molecular reaction coordinate simultaneously with thermodynamics and kinetics. Nat. Commun..

[20] Neupane K, Yu H, Foster DA, Wang F, Woodside MT (2011). Single-molecule force spectroscopy of the add adenine riboswitch relates folding to regulatory mechanism. Nucl. Acid. Res..

[21] Goodfellow, I., Bengio, Y. & Courville, A. *Deep Learning* (MIT press, 2016).

[22] Cover, T. M. & Thomas, J. A. *Elements of Information Theory* (John Wiley & Sons, 2012).

[23] Pressé S, Ghosh K, Lee J, Dill KA (2013). Principles of maximum entropy and maximum caliber in statistical physics. Rev. Mod. Phys..

[24] Moore CC (2015). Ergodic theorem, ergodic theory, and statistical mechanics. Proc. Natl Acad. Sci. USA.

[25] Noe F, Banisch R, Clementi C (2016). Commute maps: separating slowly mixing molecular configurations for kinetic modeling. J. Chem. Theor. Comp..

[26] Noé F, Clementi C (2015). Kinetic distance and kinetic maps from molecular dynamics simulation. J. Chem. Theor. Comp..

[27] Tsai ST, Tiwary P (2020). On the distance between A and B in molecular configuration space. Mol. Sim..

[28] Hänggi P, Talkner P, Borkovec M (1990). Reaction-rate theory: fifty years after kramers. Rev. Mod. Phys..

[29] Berne BJ, Borkovec M, Straub JE (1988). Classical and modern methods in reaction rate theory. J. Phys. Chem..

[30] Valsson O, Tiwary P, Parrinello M (2016). Enhancing important fluctuations: rare events and metadynamics from a conceptual viewpoint. Ann. Rev. Phys. Chem..

[31] Salvalaglio M, Tiwary P, Parrinello M (2014). Assessing the reliability of the dynamics reconstructed from metadynamics. J. Chem. Theor. Comp..

[32] Ma A, Dinner AR (2005). Automatic method for identifying reaction coordinates in complex systems. J. Phys. Chem. B.

[33] Bolhuis PG, Dellago C, Chandler D (2000). Reaction coordinates of biomolecular isomerization. Proc. Natl Acad. Sci. USA.

[34] Smith Z, Pramanik D, Tsai S-T, Tiwary P (2018). Multi-dimensional spectral gap optimization of order parameters (sgoop) through conditional probability factorization. J. Chem. Phys..

[35] Husic BE, Pande VS (2018). Markov state models: from an art to a science. J. Am. Chem. Soc..

[36] Eddy SR (2004). What is a hidden markov model?. Nat. Biotechnol..

[37] McKinney SA, Joo C, Ha T (2006). Analysis of single-molecule fret trajectories using hidden markov modeling. Bioph. Jour..

[38] Blanco, M. & Walter, N. G. Analysis of complex single-molecule fret time trajectories. In *Methods in Enzymology*, Vol. 472, 153–178 (Elsevier, 2010). 10.1016/S0076-6879(10)72011-5 PMC3012381 20580964

[39] Bowman GR, Beauchamp KA, Boxer G, Pande VS (2009). Progress and challenges in the automated construction of markov state models for full protein systems. J. Chem. Phys..

[40] Scherer MK (2015). Pyemma 2: a software package for estimation, validation, and analysis of markov models. J. Chem. Theor. Comp..

[41] Pérez-Hernández G, Paul F, Giorgino T, De Fabritiis G, Noé F (2013). Identification of slow molecular order parameters for markov model construction. J. Chem. Phys..

[42] Chodera JD, Noé F (2014). Markov state models of biomolecular conformational dynamics. Curr. Op. Struc. Bio. y..

[43] Mikolov, T., Sutskever, I., Chen, K., Corrado, G. S. & Dean, J. Distributed representations of words and phrases and their compositionality. In *Advances in Neural Information Processing Systems.* 3111–3119 (2013).

[44] Bussi G, Parrinello M (2007). Accurate sampling using langevin dynamics. Phys. Rev. E.

[45] Berendsen HJ, van der Spoel D, van Drunen R (1995). Gromacs: a message-passing parallel molecular dynamics implementation. Comp. Phys. Commun..

[46] Abraham MJ (2015). Gromacs: high performance molecular simulations through multi-level parallelism from laptops to supercomputers. SoftwareX.

[47] Bonomi M, Bussi G, Camilloni CC (2019). Promoting transparency and reproducibility in enhanced molecular simulations. Nat. Methods.

[48] Bussi G, Donadio D, Parrinello M (2007). Canonical sampling through velocity rescaling. J. Chem. Phys..

