# Supplementary material for: Learning molecular dynamics with simple language model built upon long short-term memory neural network
Source: Nat Commun. 2020 Oct 9;11:5115. doi: 10.1038/s41467-020-18959-8 (PMC7547727; doi:10.1038/s41467-020-18959-8)
Supplement: Supplementary file 1 — Supplementary Information [file 41467_2020_18959_MOESM1_ESM.pdf]

Supplementary Information: Learning Molecular Dynamics with Simple Language Model built upon Long Short-Term Memory Neural Network

Supplementary Note 1: Approximating the loss function

In the following derivation, we will show how we obtain J' introduced in Eq. 10 of main text as an estimate of minimizing cross entropy J from Eq. 9 of main text. To begin with, we start with the cross entropy J:

$$\begin{aligned} J &= -\sum_{t=0}^{T-1} \mathbf{y}^{(t)} \cdot \ln \hat{\mathbf{y}}^{(t)} \\ &= -\sum_{t=0}^{T-1} \sum_{\mathbf{s}^{(t+1)}} P(\mathbf{s}^{(t+1)} | \mathbf{s}^{(t)}, ...\mathbf{s}^{(0)}) \ln \hat{\mathbf{y}}^{(t)} \end{aligned} \tag{1}$$

where $P(\mathbf{s}^{(t+1)}|\mathbf{s}^{(t)}, ...\mathbf{s}^{(0)})$ is the conditional probability of the physical system computed from the one-hot vectors of the data. Even if the trajectory has dependency on its long-term history, as long as trajectory length $T \gg 0$, we can approximate Eq. 1 as:

$$J \approx -\sum_{t=0}^{T-1} \sum_{\mathbf{s}^{(t+1)}} \Pr(\mathbf{s}^{(t+1)} | \mathbf{s}^{(t)}, ...\mathbf{s}^{(t-T)}) \ln \hat{\mathbf{y}}^{(t)} \tag{2}$$

by letting $\mathbf{s}^{(m)} = 0$ for all negative m. As is typical in character-level language models[1], we assume the embedding dimension M is much greater than the input dimension N and rewrite the above equation as:

$$J = T \left[-\frac{1}{T} \sum_{t=0}^{T-1} \sum_{\mathbf{x}^{(t+1)}} P(\mathbf{x}^{(t+1)} | \mathbf{x}^{(t)}, ...\mathbf{x}^{(t-T+1)}) \ln Q(\mathbf{x}^{(t+1)} | \mathbf{x}^{(t)}, ...\mathbf{x}^{(t-T)}) \right] \tag{3}$$

$$= T \left[\frac{1}{T} \sum_{t=0}^{T-1} \tilde{J}^{(t)}(\mathbf{x}^{(t)}, ...\mathbf{x}^{(t-T+1)}) \right] \tag{4}$$

where $\tilde{J}^{(t)}(\mathbf{x}^{(t)}, ...\mathbf{x}^{(t-T+1)}) \equiv -\sum_{\mathbf{x}^{(t+1)}} P(\mathbf{x}^{(t+1)}|\mathbf{x}^{(t)}, ...\mathbf{x}^{(t-T+1)}) \ln Q(\mathbf{x}^{(t+1)}|\mathbf{x}^{(t)}, ...\mathbf{x}^{(t-T+1)})$ is the cross entropy between conditional probabilities. With large enough T, we can also

assume ergodicity and convert the time average to ensemble average,

$$J \approx T \sum_{\mathbf{x}^{(T-1)}\ldots\mathbf{x}^{(0)}} P(\mathbf{x}^{(T-1)},...,\mathbf{x}^{(0)})\bar{J}(\mathbf{x}^{(T-1)},...\mathbf{x}^{(0)}) \tag{5}$$

$$= -T\sum_{\mathbf{x}^{(T)}} \sum_{\mathbf{x}^{(T-1)},\mathbf{x}^{(T-2)}\ldots\mathbf{x}^{(0)}} P(\mathbf{x}^{(T-1)},...,\mathbf{x}^{(0)})P(\mathbf{x}^{(T)}|\mathbf{x}^{(T-1)}...,\mathbf{x}^{(0)}) \ln Q(\mathbf{x}^{(T)}|\mathbf{x}^{(T-1)}...,\mathbf{x}^{(0)}) \tag{6}$$

$$= -T \sum_{\mathbf{x}^{(T)}\ldots\mathbf{x}^{(0)}} P(\mathbf{x}^{(T)},...,\mathbf{x}^{(0)}) \ln Q(\mathbf{x}^{(T)}|\mathbf{x}^{(T-1)}...,\mathbf{x}^{(0)}) \tag{7}$$

The cross entropy J achieves its global minima when Q approaches P :

$$Q(\mathbf{x}^{(t+1)}|\mathbf{x}^{(t)},...\mathbf{x}^{(t-T+1)}) \rightarrow P(\mathbf{x}^{(t+1)}|\mathbf{x}^{(t)},...\mathbf{x}^{(t-T+1)}) \quad \forall\, t \tag{8}$$

We also know that

$$Q(\mathbf{x}^{(T)}|\mathbf{x}^{(T-1)},...,\mathbf{x}^{(0)}) = \frac{Q(\mathbf{x}^{(T)},...,\mathbf{x}^{(0)})}{Q(\mathbf{x}^{(T-1)},...,\mathbf{x}^{(0)})} \tag{9}$$

Plugging Eq. 9 in Eq. 7,

$$\begin{aligned}
J &= -T \sum_{\mathbf{x}^{(T)}\ldots\mathbf{x}^{(0)}} P(\mathbf{x}^{(T)},...,\mathbf{x}^{(0)}) \ln Q(\mathbf{x}^{(T)}|\mathbf{x}^{T-1},...,\mathbf{x}^{(0)}) \\
&= -T \sum_{\mathbf{x}^{(T)}\ldots\mathbf{x}^{(0)}} \left[P(\mathbf{x}^{(T)},...,\mathbf{x}^{(0)}) \ln Q(\mathbf{x}^{(T)},...,\mathbf{x}^{(0)}) - P(\mathbf{x}^{(T)},...,\mathbf{x}^{(0)}) \ln Q(\mathbf{x}^{(T-1)},...,\mathbf{x}^{(0)})\right] \\
&= -T \sum_{\mathbf{x}^{(T)}\ldots\mathbf{x}^{(0)}} P(\mathbf{x}^{(T)},...,\mathbf{x}^{(0)}) \ln Q(\mathbf{x}^{(T)},...,\mathbf{x}^{(0)}) + T \sum_{\mathbf{x}^{(T)}\ldots\mathbf{x}^{(0)}} P(\mathbf{x}^{(T)},...,\mathbf{x}^{(0)}) \ln Q(\mathbf{x}^{(T-1)},...,\mathbf{x}^{(0)}) \\
&= J' - J''
\end{aligned}$$

where $Q(\mathbf{x}^{(T)},...,\mathbf{x}^{(0)}) \rightarrow P(\mathbf{x}^{(T)},...,\mathbf{x}^{(0)})$ during the minimization. Here we have defined J' and J'' as follows:

$$J' \equiv -T \sum_{\mathbf{x}^{(T)}\ldots\mathbf{x}^{(0)}} P(\mathbf{x}^{(T)},...,\mathbf{x}^{(0)}) \ln Q(\mathbf{x}^{(T)},...,\mathbf{x}^{(0)}) \tag{10}$$

$$J'' \equiv -T \sum_{\mathbf{x}^{(T)}\ldots\mathbf{x}^{(0)}} P(\mathbf{x}^{(T)},...,\mathbf{x}^{(0)}) \ln Q(\mathbf{x}^{(T-1)},...,\mathbf{x}^{(0)}) \tag{11}$$

$$= -T \sum_{\mathbf{x}^{(T-1)}\ldots\mathbf{x}^{(0)}} P(\mathbf{x}^{(T-1)},...,\mathbf{x}^{(0)}) \ln Q(\mathbf{x}^{(T-1)},...,\mathbf{x}^{(0)}) \tag{12}$$

Since the summations run over the state space such that the normalization condition of P and Q holds, according to Gibbs' inequality J' and J'' both are well-defined cross entropies, and their global minima happen when $Q = P$. Therefore, minimizing J leads to minimization of both J' and J'', at which point both J' and J'' are path entropies.

Alanine dipeptide			
CVs	Label	C_{7eq} to C_{7ax} (ps)	C_{7ax} to C_{7eq} (ps)
$\sin\phi$	actual	5017.33 ± 966.714	106.63± 20.284
	LSTM	6098.78 ± 1084.492	125.90 ± 25.779
$\sin\psi$	actual	5169.94 ± 608.618	115.98 ± 19.883
	LSTM	5050.85 ± 640.132	97.06 ± 13.519

Supplementary Table 1 Inverse of transition rates with stochastic gradient optimization run for 60 training epochs.

Supplementary Note 2: Training alanine dipeptide for more epochs

Supplementary Table 1 shows the calculated transition rates for conformational change in alanine dipeptide when running stochastic gradient optimization for 60 training epochs. The result is similar to what we have for 40 epochs.

Supplementary Note 3: Comparing computational effort

In this section, we compare the computational effort (as measured through wall-clock time) in training/estimating LSTM/MSM/HMM and simulating trajectory data. In Supplementary Table 2, we have shown the effort for LSTM when analyzing each model potential in a single run. In Supplementary Table 3, we have shown the efforts for MSM and HMM needed to set the lag time τ and the time for running the Chapman-Kolmogorov (CK) test.

Model	System	Model training (s)	Simulate data (s)
LSTM	linear 3-state	103	53.4
	triangular 3-state	103	213.7
	4-state	183	642.0

Supplementary Table 2 Computational effort for a LSTM run.

Model	τ	Find t_I (s)	CK test (s)	Model building (s)	Simulate data (s)
MSM	1	23.9±0.08	32.6±0.36	3.0±0.04	84.4±3.3
	5		35.6±0.36	2.9±0.04	15.4±0.22
HMM	1	48.6±1.89	597.0±7.39	79.4±1.34	290.6±4.01
	5		134.2±1.00	157.2±0.52	57.8±0.77

Supplementary Table 3 Computational efforts for MSM and HMM when analyzing trajectories from model systems. τ and t_I are the lag time used for building the models and the implied timescale of the system. The recorded times are the sum for linear 3-state, triangular 3-state, and 4-state. The results are averaged over 5 independent runs.

Supplementary Note 4: The kinetics learned by MSM and HMM

In this section, we reproduce all calculations we have done for LSTM with MSM and HMM. The implied timescale analysis are first shown in Supplementary Figure 1 for MSM and Supplementary Figure 2 for HMM. After that, the commitment time analysis for 3-state and 4-state are shown respectively (Supplementary Figure 3-10). Finally we show the analysis of experimental trajectory: The implied timescale analyses for MSM and HMM using 21 hidden states are shown in Supplementary Figure 11. The corresponding commitment time analysis are shown in Supplementary Figure 13. For the HMM with 3 hidden states shown in the main text, we perform both the implied timescale analysis and the CK test with lag time=$3ms$ in Supplementary Figure 11 and 12. Note that all the following analysis are performed with smoothened binned trajectories in order to compare with our results in the main text directly. The analysis with unsmoothened data is put in the final section of this file (please see Supplementary Note 6 and Supplementary Figs. 19-22).

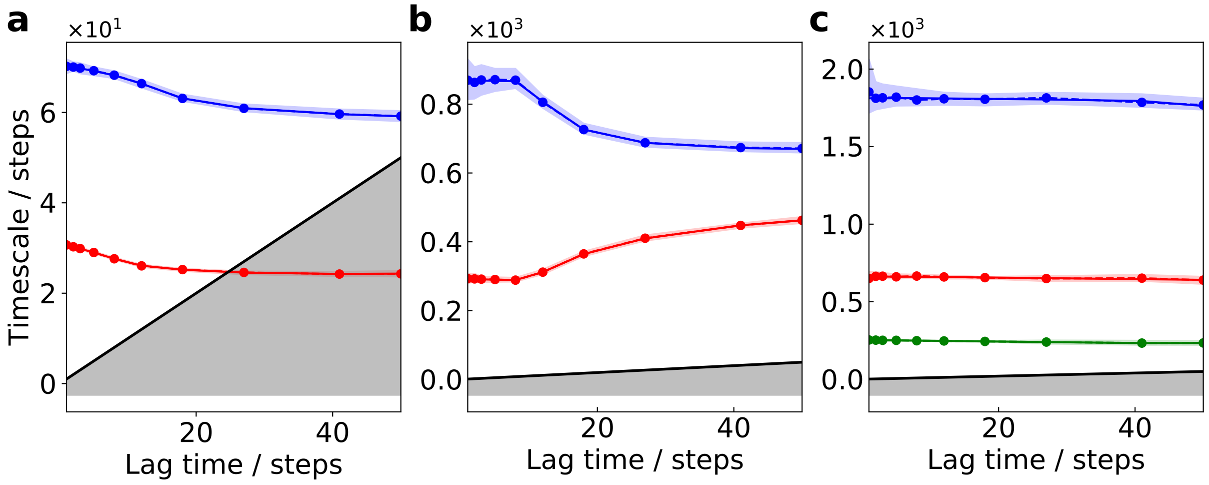

Supplementary Figure 1 The implied timescale versus lag time of MSM for **a** linear 3-state, **b** triangular 3-state, and **c** 4-state model potentials. The color blue, red, and green represent the first, second, third slowest implied timescales calculated from the eigenvalues. The markers connected by the dashed lines denote the mean, and the region filled with the color represents their 95% confidence interval. The solid lines are the corresponding maximum likelihood estimates. The black solid line indicates the symmetric $x = y$ line.

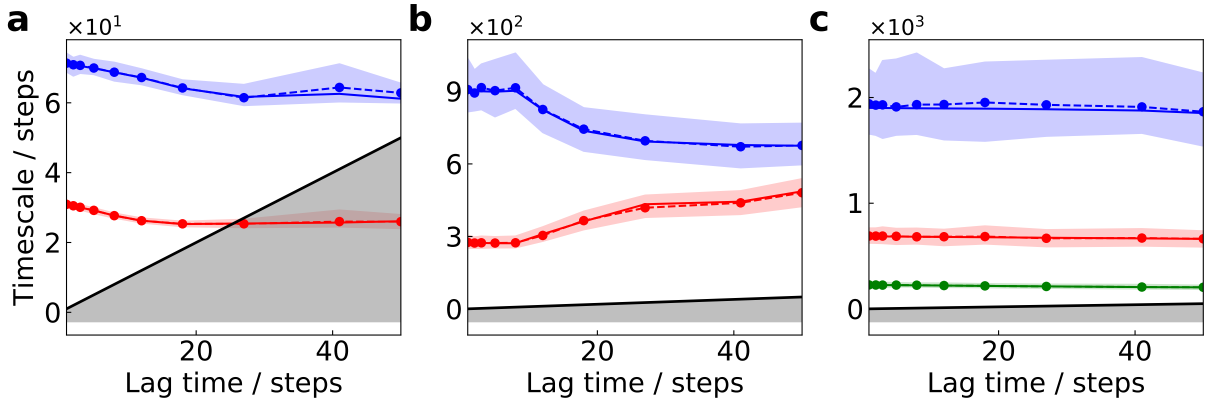

Supplementary Figure 2 The implied timescale versus lag time of HMM for **a** linear 3-state, **b** triangular 3-state, and **c** 4-state model potentials. The color blue, red, and green represent the first, second, third slowest implied timescales calculated from the eigenvalues. The markers connected by the dashed lines denote the mean, and the region filled with the color represents their 95% confidence interval. The solid lines are the corresponding maximum likelihood estimates. The black solid line indicates the symmetric $x = y$ line.

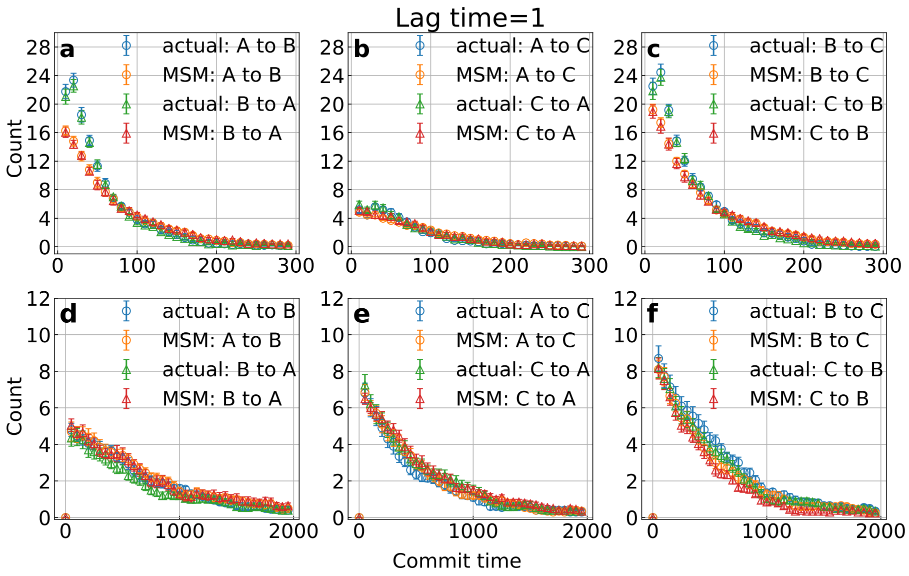

Supplementary Figure 3 Number of transitions between different pairs of metastable states as a function of commitment time defined in the main text. The calculations for linear and triangular configurations are shown in **a-c** and **d-f** respectively. Error bars are illustrated and were calculated as standard errors.

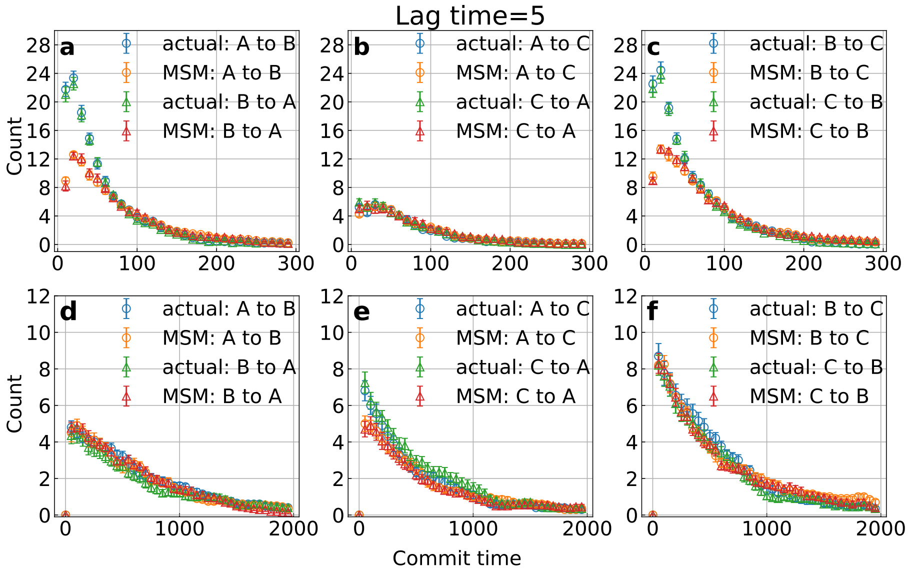

Supplementary Figure 4 Number of transitions between different pairs of metastable states as a function of commitment time defined in the main text. The calculations for linear and triangular configurations are shown in **a-c** and **d-f** respectively. Error bars are illustrated and were calculated as standard errors.

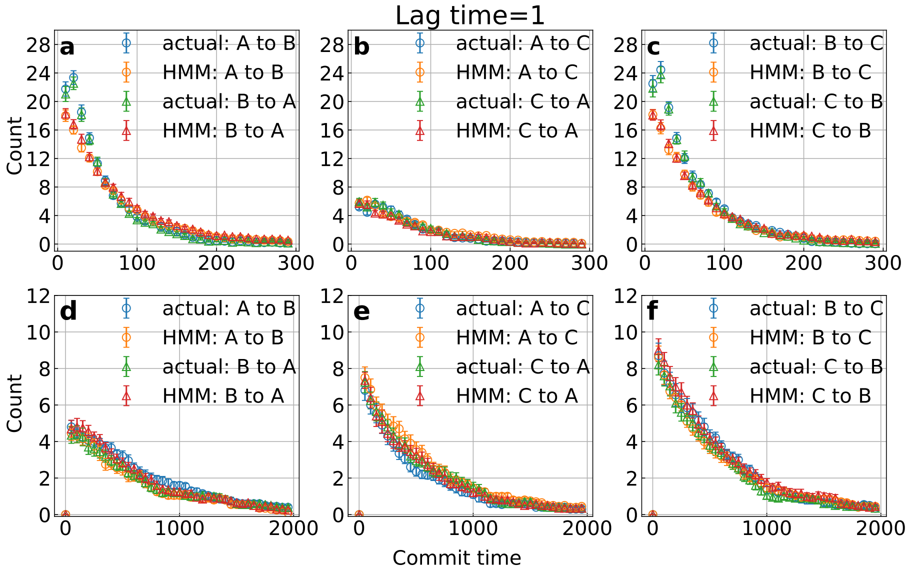

Supplementary Figure 5 Number of transitions between different pairs of metastable states as a function of commitment time defined in the main text. The calculations for linear and triangular configurations are shown in **a-c** and **d-f** respectively. Error bars are illustrated and were calculated as standard errors.

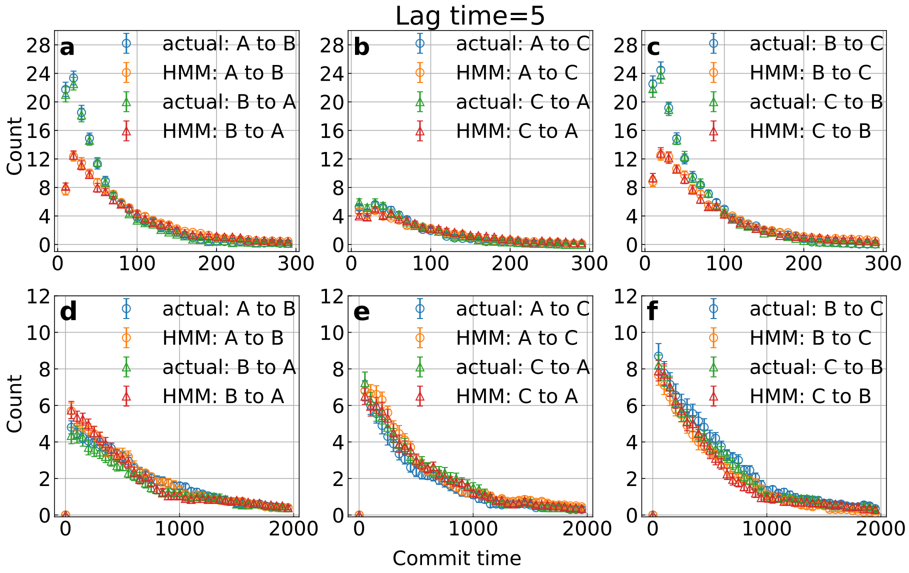

Supplementary Figure 6 Number of transitions between different pairs of metastable states as a function of commitment time defined in the main text. The calculations for linear and triangular configurations are shown in **a-c** and **d-f** respectively. Error bars are illustrated and were calculated as standard errors.

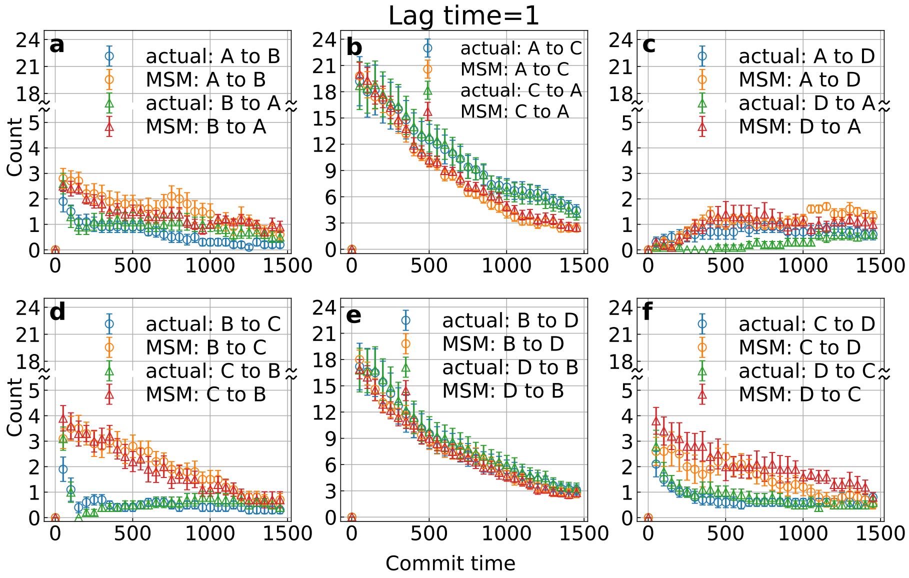

Supplementary Figure 7 Number of transitions between different pairs of metastable states as a function of commitment time defined in the main text for 4-state model system. Error bars are illustrated and were calculated as standard errors.

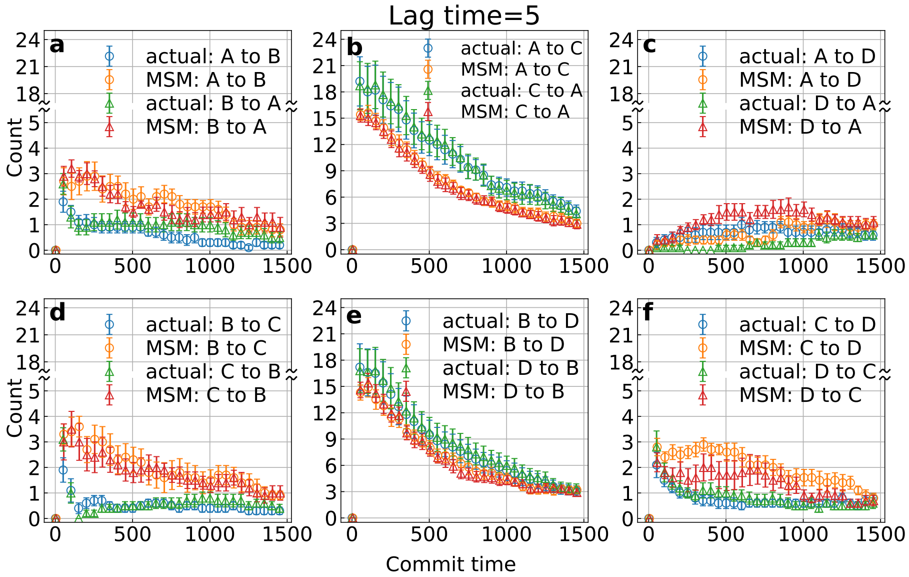

Supplementary Figure 8 Number of transitions between different pairs of metastable states as a function of commitment time defined in the main text for 4-state model system. Error bars are illustrated and were calculated as standard errors.

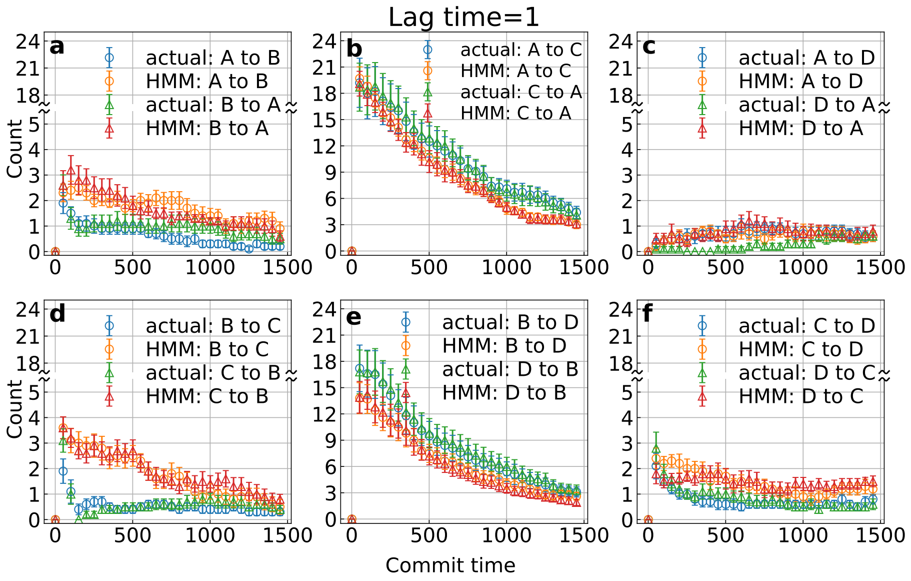

Supplementary Figure 9 Number of transitions between different pairs of metastable states as a function of commitment time defined in the main text for 4-state model system. Error bars are illustrated and were calculated as standard errors.

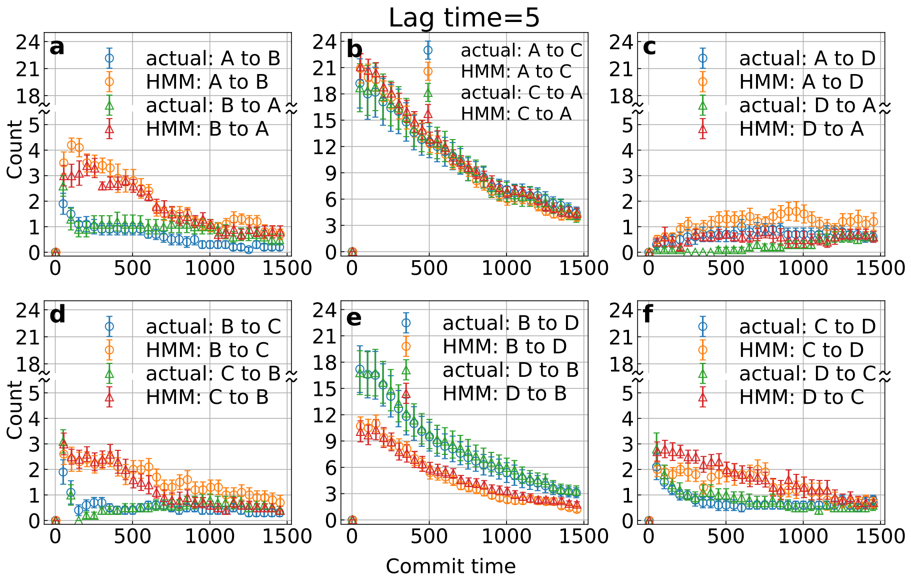

Supplementary Figure 10 Number of transitions between different pairs of metastable states as a function of commitment time defined in the main text for 4-state model system. Error bars are illustrated and were calculated as standard errors.

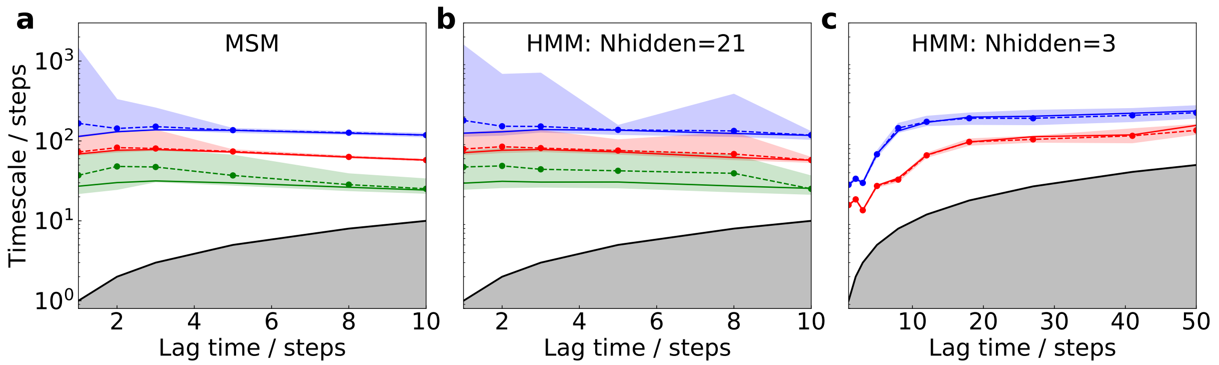

Supplementary Figure 11 The implied timescale analysis of **a** MSM **b** HMM with 21 hidden states **c** HMM with 3 hidden states built from the single-molecule FRET experiment trajectory. Since the data is collected at 10kHz, each unit step corresponds to 0.1 ms. The color blue, red, and green represent the first, second, third slowest implied timescales calculated from the eigenvalues. The markers connected by the dashed lines denote the mean, and the region filled with the color represents their 95% confidence interval. The solid lines are the corresponding maximum likelihood estimates. The black solid line indicates the symmetric $x = y$ line.

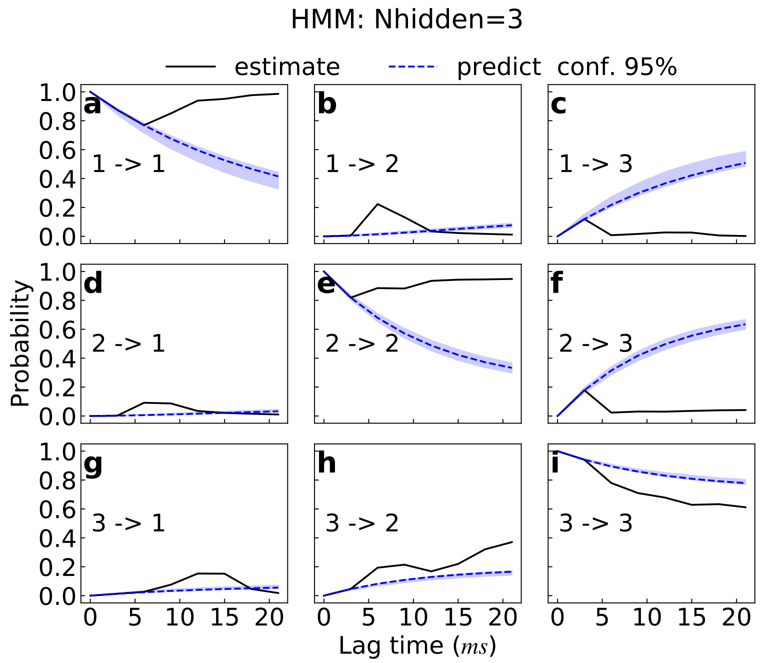

Supplementary Figure 12 The Chapman-Kolmogorow (CK) test of HMM built from the single-molecule FRET experiment trajectory. The number of hidden states used equals 3. The lag time used for CK test is $3ms$. The region filled with blue is the 95% confidence interval.

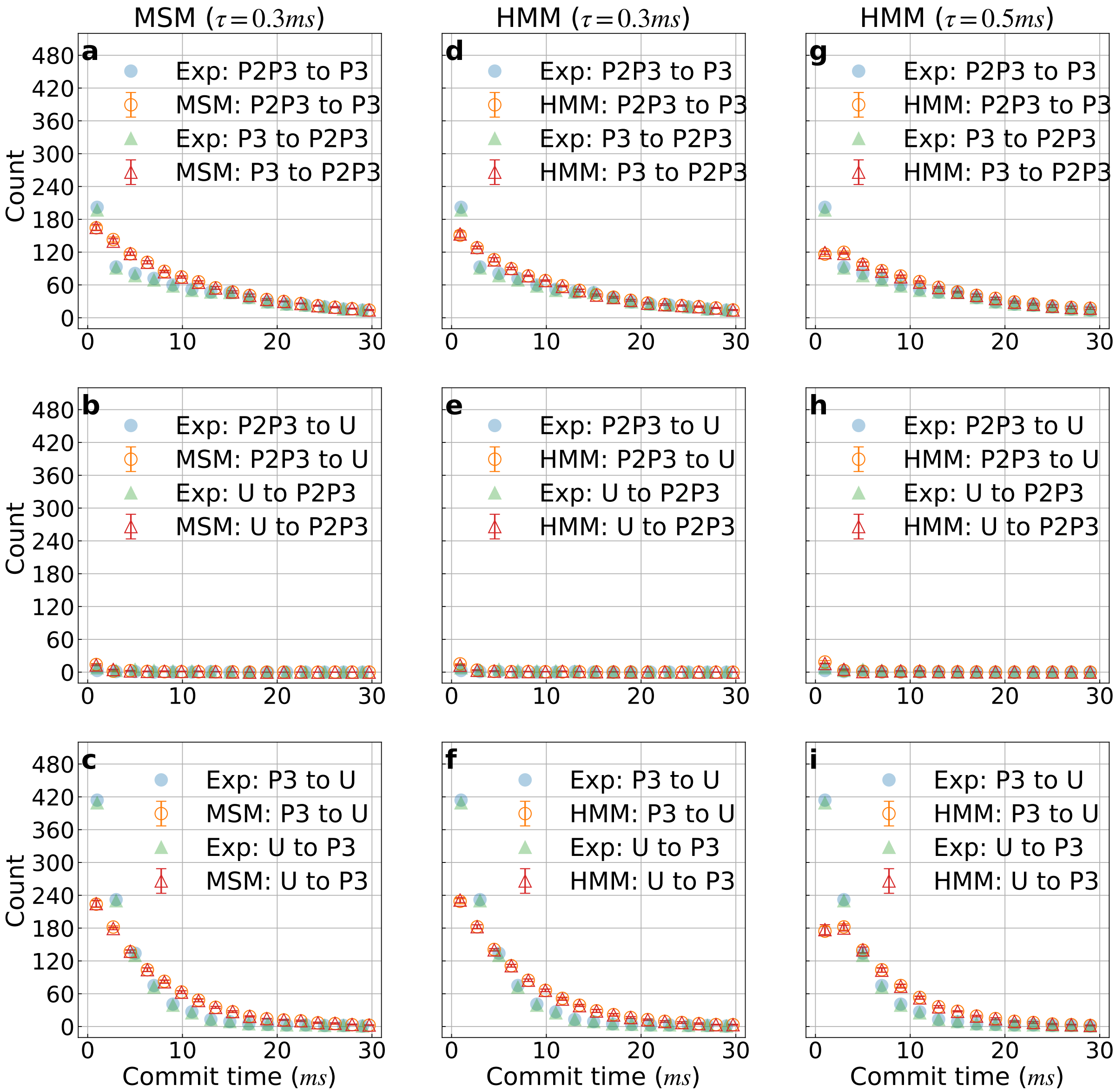

Supplementary Figure 13 Number of transitions between different pairs of metastable states as a function of commitment time defined in for the experimental data learned by MSM and HMM. The models were built with lag time=$0.3ms$. The number of hidden states used for HMM is 21. Error bars are illustrated and were calculated as standard errors.

Supplementary Note 5: Trajectories

In this section we provide representative trajectories obtained for the different systems described in the main text, where Supplementary Figure 14 is for linear 3-state model potential, Supplementary Figure 15 is for triangular 3-state model potential, Supplementary Figure 16 is for 4-state model potential, Supplementary Figure 17 is for alanine dipeptide, and Supplementary Figure 18 is for force spectroscopy trajectory. We also show how we removed ephemeral/spurious transitions by smoothening the binned trajectory before feeding into the LSTM model, in order to make the learning process more stable. The emphemoral/spurious transitions happened due to the use of only 3 labels for 3-state model systems and 4 labels for 4-state model system when projecting on the x-axis. It is not related to the learning quality of LSTM. With more labels or finer binning, we can avoid producing those spurious states and train the LSTM without smoothening binned trajectory before feeding into the model. This can be seen from the example of alanine dipeptide (Supplementary Figure 17), where we did not smoothen the trajectory.

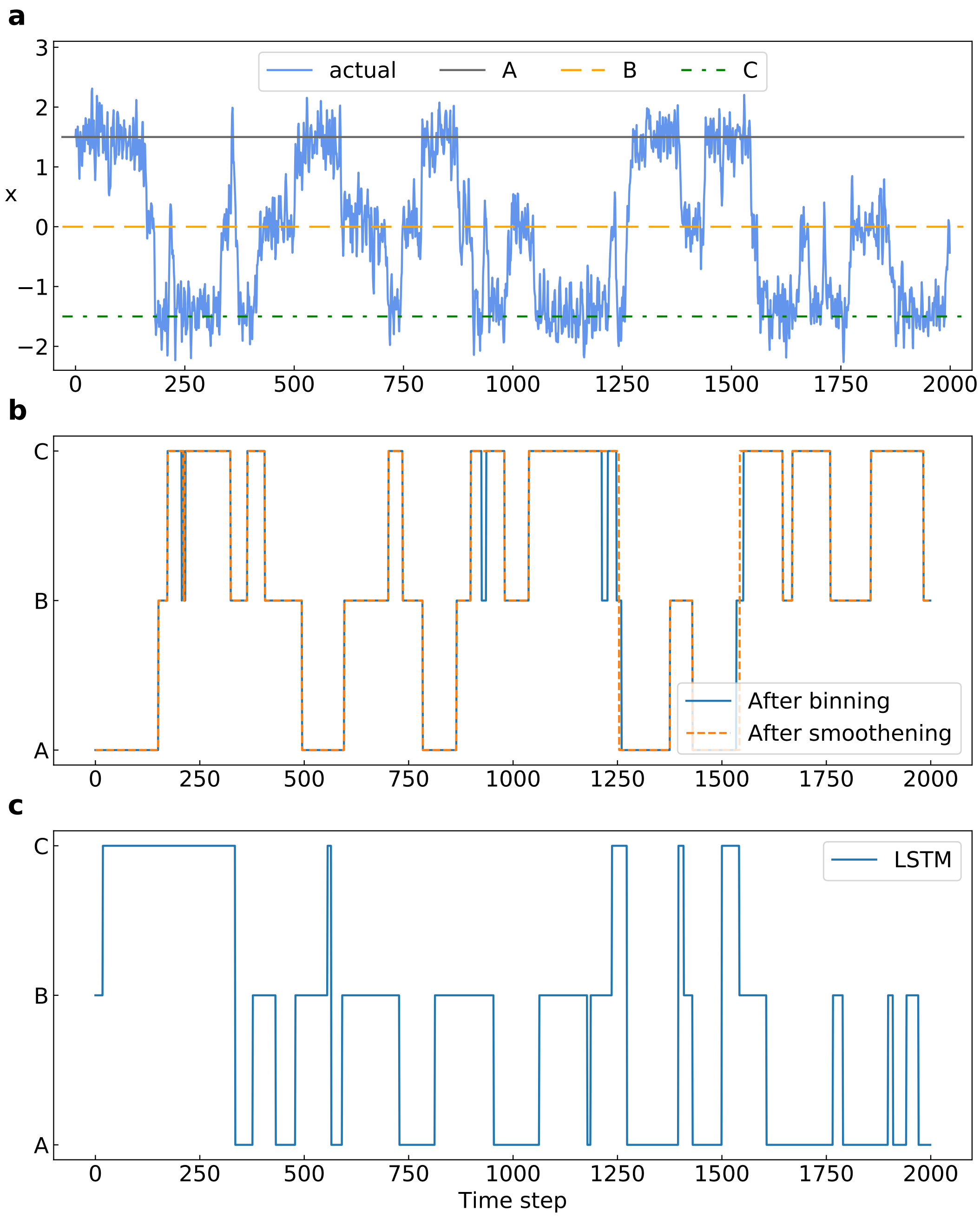

Supplementary Figure 14 Trajectories for linear 3-state model potential. **a** The actual trajectory with location of metastable states shown by horizontal solid and dashed lines. **b** The trajectory after spatial discretization, where the trajectory now consists of a sequence of labels representing metastable states. To make the learning process more stable, we removed ephemeral/spurious transitions by smoothening before feeding into the LSTM model. **c** The trajectory generated by our LSTM model.

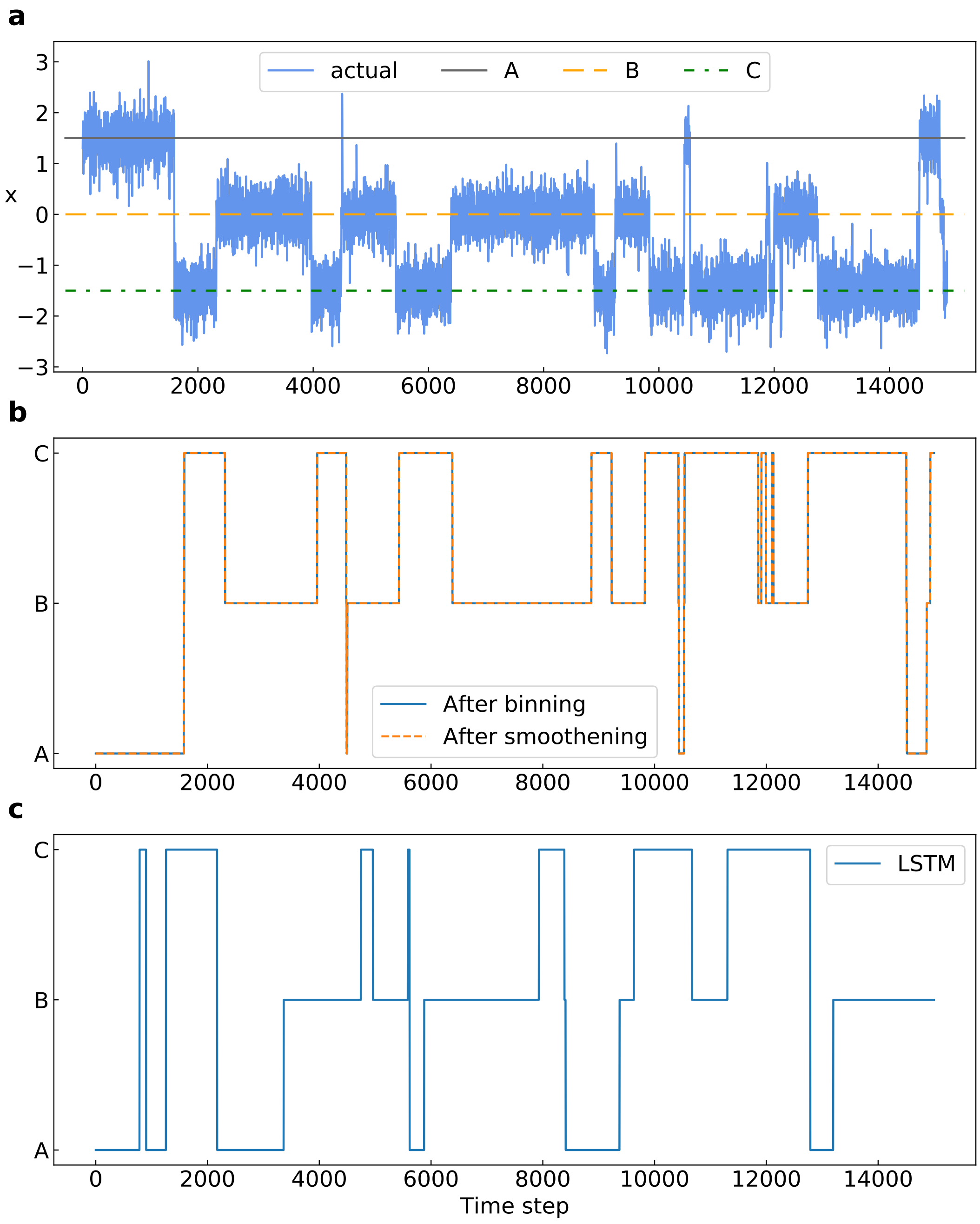

Supplementary Figure 15 Trajectories for triangular 3-state model potential. **a** The actual trajectory with location of metastable states shown by horizontal solid and dashed lines. **b** The trajectory after spatial discretization, where the trajectory now consists of a sequence of labels representing metastable states. To make the learning process more stable, we removed ephemeral/spurious transitions by smoothening before feeding into the LSTM model. **c** The trajectory generated by our LSTM model.

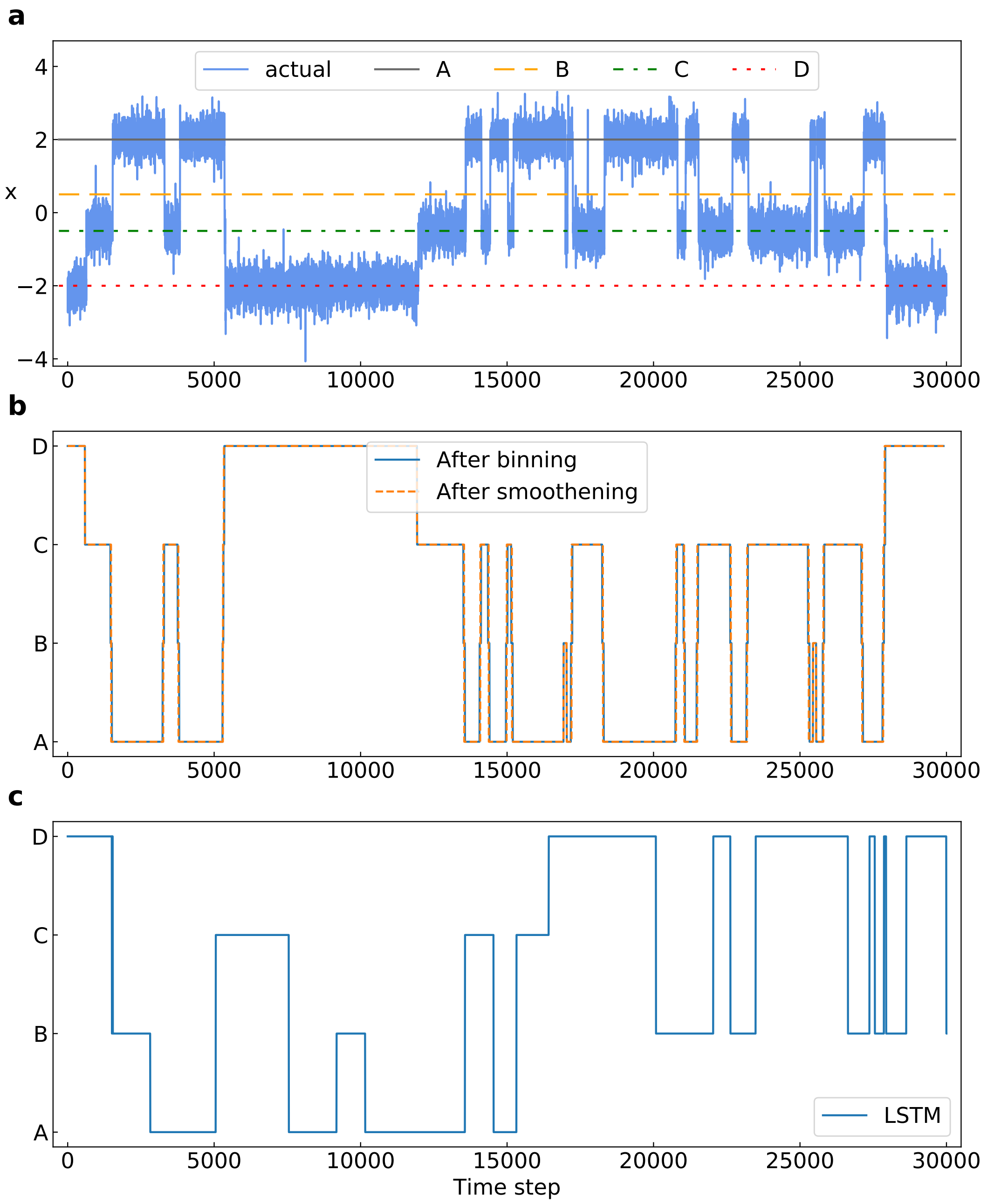

Supplementary Figure 16 Trajectories for 4-state model potential. **a** The actual trajectory with location of metastable states shown by horizontal solid and dashed lines. **b** The trajectory after spatial discretization, where the trajectory now consists of a sequence of labels representing metastable states. To make the learning process more stable, we removed ephemeral/spurious transitions by smoothening before feeding into the LSTM model. **c** The trajectory generated by our LSTM model.

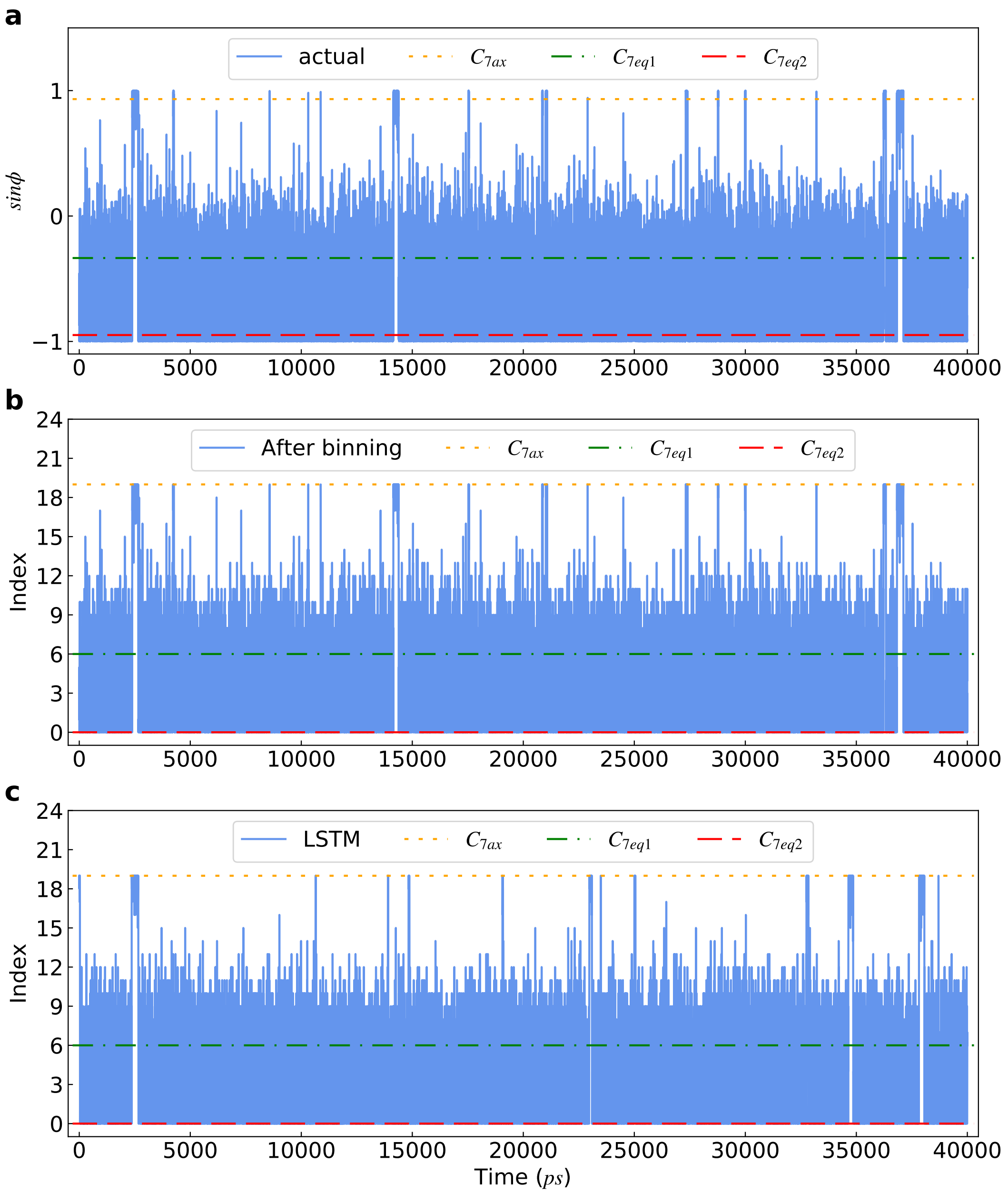

Supplementary Figure 17 Trajectories along $\sin\phi$ for alanine dipeptide. **a** The actual MD trajectory with location of metastable states shown by horizontal dashed lines. **b** The trajectory after spatial discretization into 20 indexed positions. **c** The predicted trajectory generated by our LSTM model.

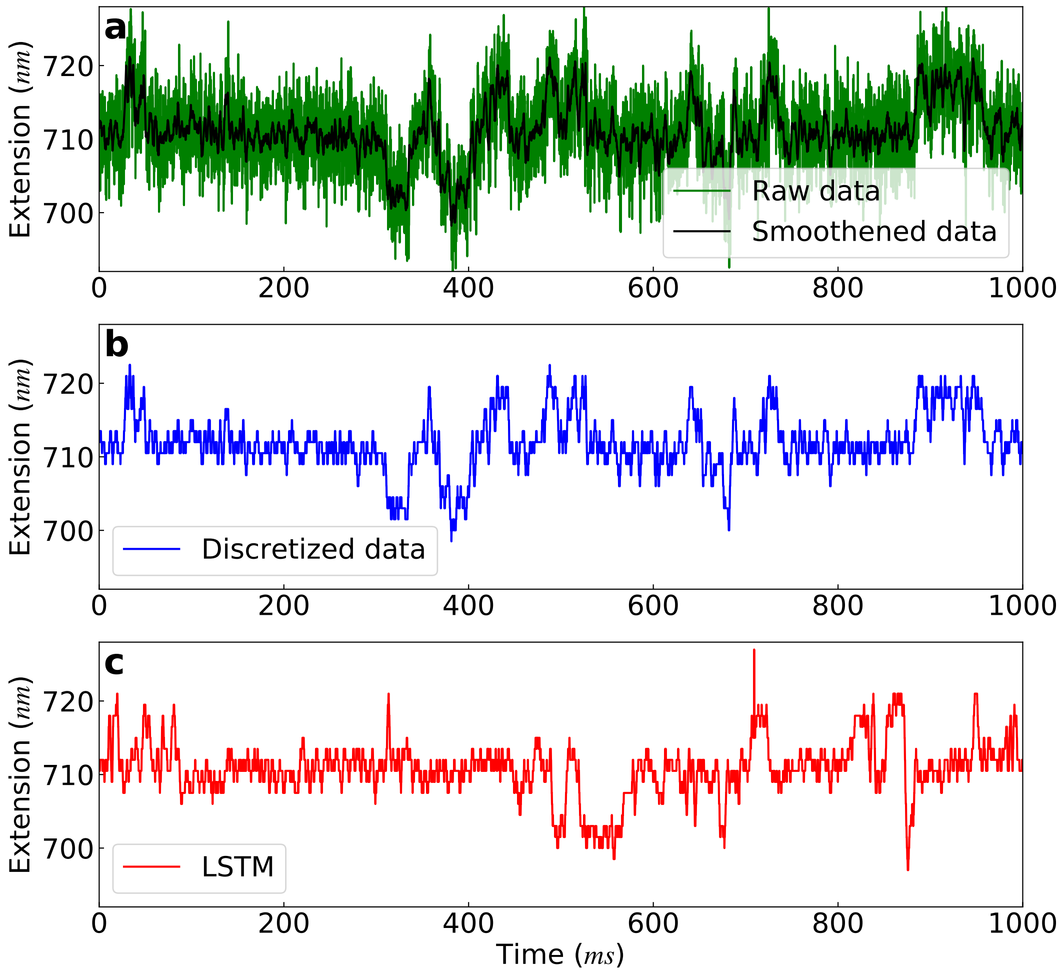

Supplementary Figure 18 Trajectories for single molecule force spectroscopy experiment on riboswitch. **a** The original trajectory and the corresponding smoothened trajectory. **b** The trajectory after spatial discretization into 34 indexed positions. **c** The predicted trajectory generated by the LSTM model.

Supplementary Note 6: Tests without smoothening data

In this section, we briefly show the analysis of Boltzmann statistics and kinetics using LSTM, MSM, and HMM. Without smoothening, it is hard to see three metastable states from the probability distribution. In addition, the trajectory stays non-Markovian even for a large lag time, which can be seen from Supplementary Figure 19. Either MSM or HMM cannot learn Boltzmann statistics and kinetics from the system, while LSTM still capture both (Supplementary Figure 21). Note that from Supplementary Figure 19**a**, we are unable to find a good lag time for MSM. At lag time/steps≈40 (or equivalently lag time=4ms), there is a plateau for HMM with 3 hidden states. However, from Supplementary Figure 21**b-d**, we can see that the count in all plots vanishes when commitment time=3ms, which means that the typical timescale for back and forth transitions is less than 3ms for unsmoothened trajectory. Therefore, we are not able to see kinetics if we make a HMM with lag time=4ms. This is as expected since the lag time is too large such that the trajectory is always Markovian.

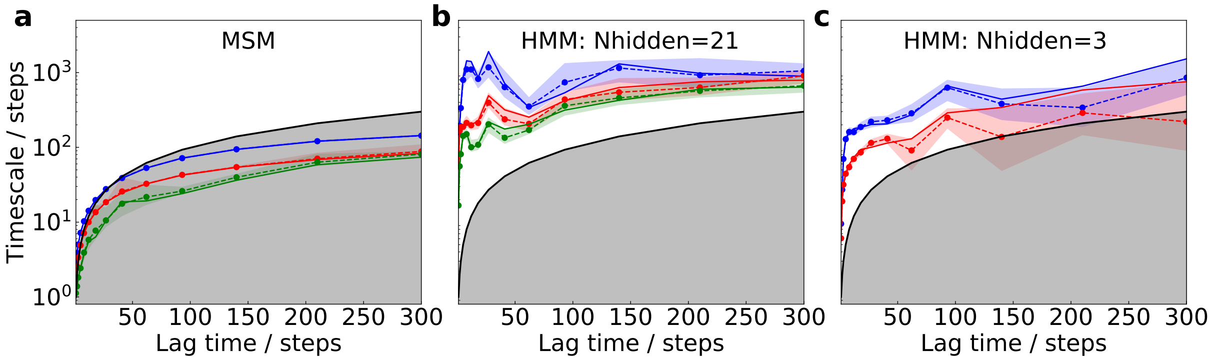

Supplementary Figure 19 The implied timescale analysis of **a** MSM **b** HMM with 21 hidden states **c** HMM with 3 hidden states. Since the data is collected at 10kHz, each unit step corresponds to 0.1 ms. The plots were obtained without smoothening the trajectory. The color blue, red, and green represent the first, second, third slowest implied timescales calculated from the eigenvalues. The markers connected by the dashed lines denote the mean, and the region filled with the color represents their 95% confidence interval. The solid lines are the corresponding maximum likelihood estimates. The black solid line indicates the symmetric $x = y$ line.

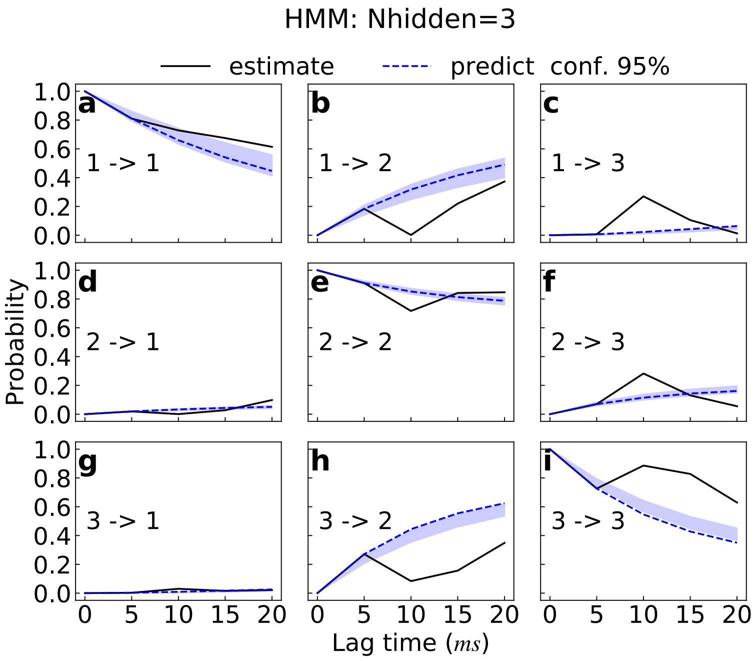

Supplementary Figure 20 The Chapman-Kolmogorow (CK) test of HMM built from the single-molecule FRET experiment trajectory. The number of hidden states used equals 3. The lag time used for CK test is $5ms$. The region filled with blue is the 95% confidence interval.

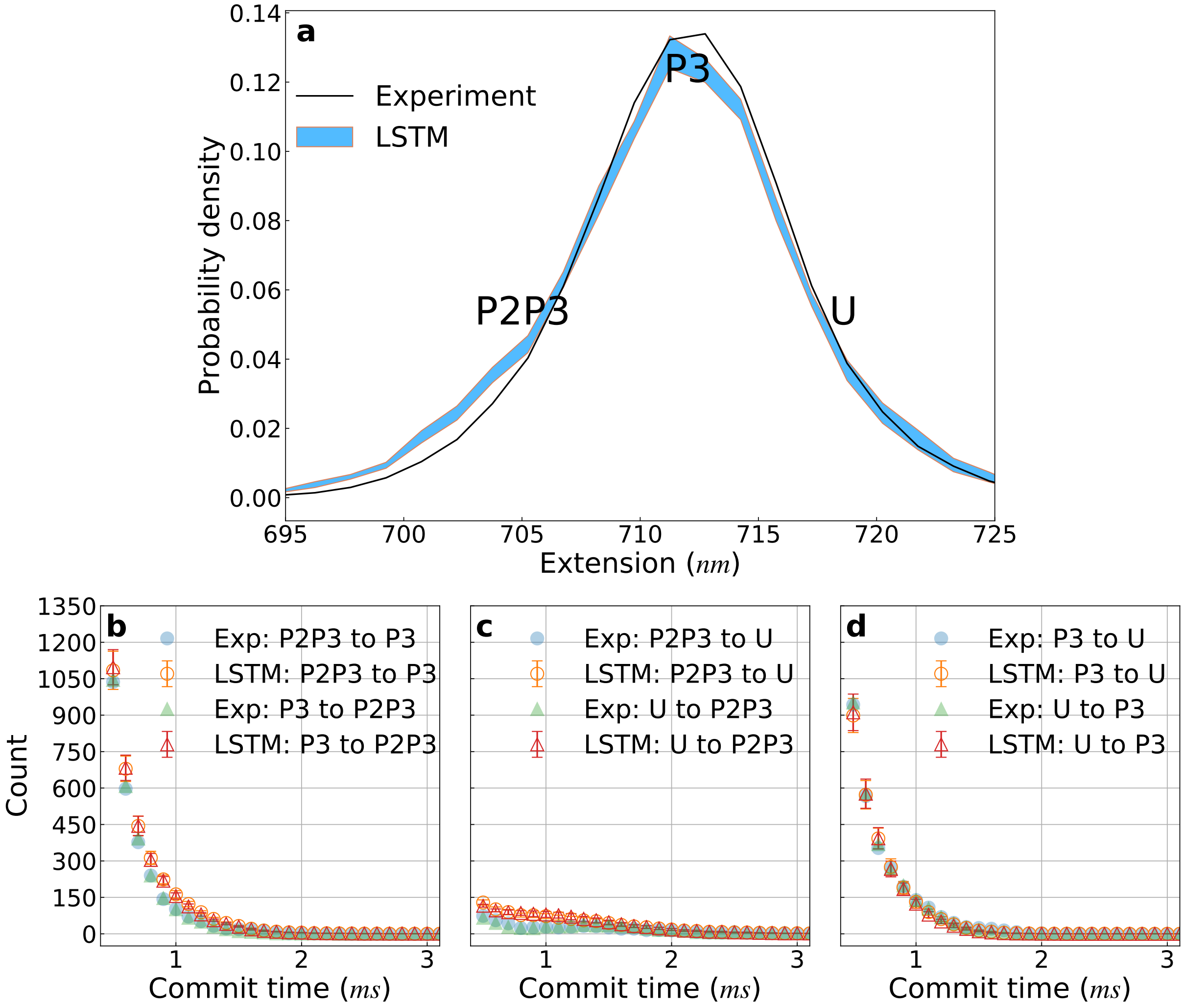

Supplementary Figure 21 In this figure, we show our result of using the LSTM model to learn the kinetics from the unsmoothened experiment trajectory. **a** A comparison between the probability density learned by the LSTM model and calculated from the experimental data **b-d** The commit time plots calculated by counting the transitions in the trajectory generated by LSTM and the experimental trajectory. Error bars are illustrated and were calculated as standard errors.

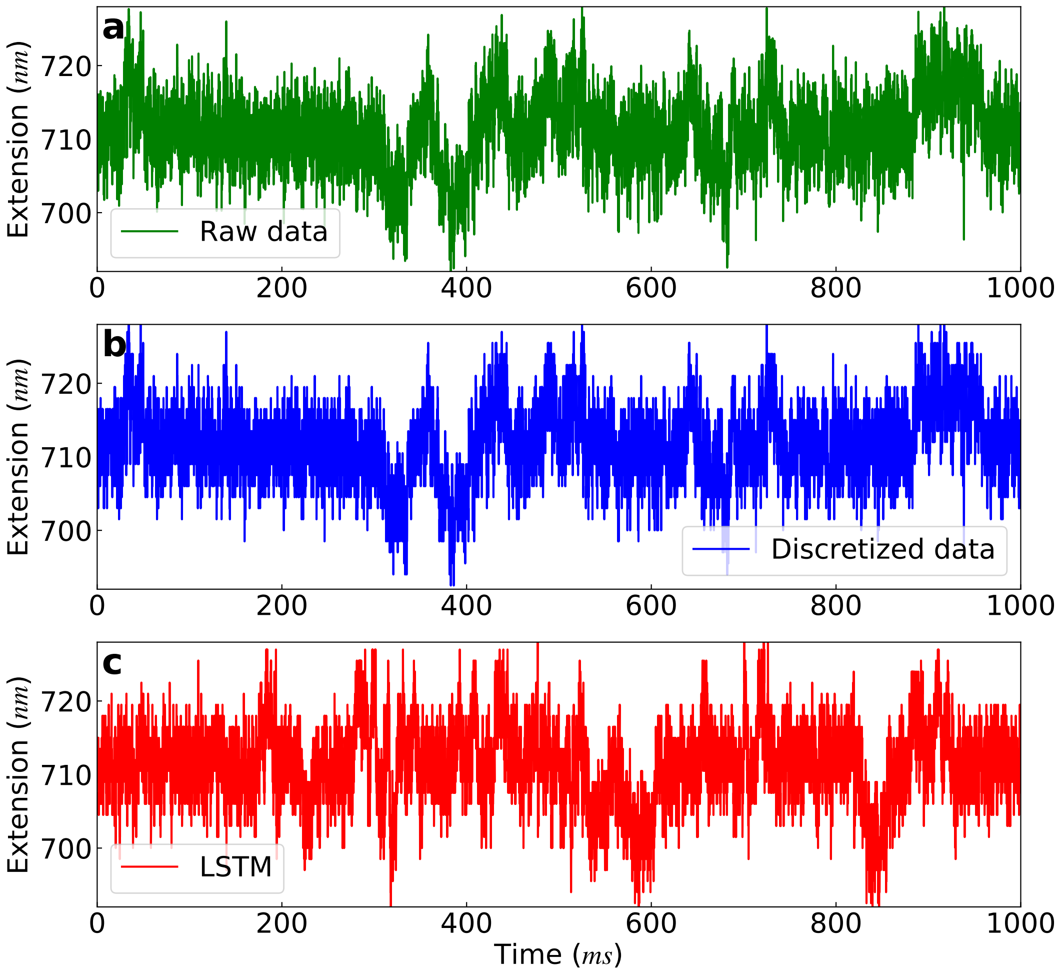

Supplementary Figure 22 Trajectories for single molecule force spectroscopy experiment on riboswitch, this time without any smoothening. **a** The actual, unsmoothened experimental trajectory. **b** The trajectory after spatial discretization into 34 indexed positions. **c** The predicted trajectory generated by the LSTM model.

Supplementary References

[1] Rami Al-Rfou, Dokook Choe, Noah Constant, Mandy Guo, and Llion Jones. Character-level language modeling with deeper self-attention. In Proceedings of the AAAI Conference on Artificial Intelligence, 33, 3159–3166 (2019).
