# Supplementary material for: Learning molecular dynamics with simple language model built upon long short-term memory neural network
Source: Nat Commun. 2020 Oct 9;11:5115. doi: 10.1038/s41467-020-18959-8 (PMC7547727; doi:10.1038/s41467-020-18959-8)
Supplement: Supplementary file 2 — Peer Review File [file 41467_2020_18959_MOESM2_ESM.pdf]

Reviewer #1 (Remarks to the Author):

Review of "Learning Molecular Dynamics with Simple Language Model built upon Long Short-Term Memory Neural Network" by Tsai, Kuo, and Tiwary.

In their manuscript, the authors apply long short-term memory (LSTM) neural networks to analyze the trajectories of stochastic dynamics. Data-driven methods, and in particular machine learning/AI methods, in this area have a broad scope of application, including the analysis of experimental and simulation data to build an understanding of the underlying physics, chemistry, or biology of complex systems.

There is a long history of literature in this field, in particular in statistical physics, and more recently also in applied machine learning. Particularly successful methods include Master equation-based methods for conformational dynamics / Markov state Models, Hidden Markov models, time-lagged Independent Component analysis. More recently, AI/ML methods such as Dynamic Graphical Models and VAMP-derived methods (Variational approach for learning Markov Processes), including VAMPnets, show great promise.

The application to LSTM to dynamical systems in the natural sciences is sound, as this architecture is tailor-made to tackle sequential data. What is particularly intriguing about LSTMs is their ability to maintain information beyond the previous state to predict the current state. In effect, this means that we can relax the assumption of first-order Markovianity of the observed dynamics, that many current methods invoke. However, it remains unclear whether this relaxation may be beneficial from a practical perspective, in particular when the only dominant dynamical modes are of interest. Further, an interpretation of the LSTM from physical point-of-view is currently lacking in the best of this readers' knowledge. This gap may be a cause of concern for members of our community who are skeptical of 'black-box' methods.

While the use of LSTMs to dynamic problems in the natural sciences appears like a straightforward endeavor, the authors go well beyond applying pre-existing AI-methodology. In particular, the authors make the following significant contributions and explicitly address some critical gaps in our understanding:

- They show that the minimization of a cross-entropy loss used to train LSTMs of natural languages corresponds to matching the cross-entropy of true trajectory path and the modeled trajectory path. In particular, they show that the path entropy of the true dynamical process bounds this cross-entropy. 'True' here refers to the process observed in data used to train the model.

- They illustrate how the learned embedding space (A linear transformation of a spatial encoding of a time-point) enter into expressions of the transition probability (under an assumption of a stationary first-order Markovianity assumption) and may capture the average commute time between two points.

- Through numerical experiments on several tractable model systems, the authors further illustrate how the LSTM accurately predicts key thermodynamic and kinetic properties of the underlying systems.

I find that the manuscript should be published. The study addresses a highly interesting topic of interest to a broad community of researchers. Overall, the study gives the impression to be well-planned and rigorously executed, and presents theoretical and computational results in a well-written and visually appropriate manner.

I only have a handful of minor suggestions that may help underline the significance of the points made in the manuscript to a broader audience:
Please compare all the results from the LSTMs to the current state of the art methods using a similar

state encoding (a discretization of a sub-optimal 1D reaction coordinate). Either Markov state models or Hidden Markov models. I think this point is particularly important as the authors themselves state in the conclusion that these methods can perform a similar task. Still, they later state that they expect LSTM may, in general, outperform traditional methods. Consequently, having traditional methods as baselines would be a helpful first step towards substantiating this statement.
It would be helpful to include an example that illustrates the analysis of an experimental time-series, or simulation data of a protein or nucleic acid system, fx from protein-folding. And again, compare these analyses state of the art analysis techniques. An example could be single-molecule FRET trajectories analyzed via HMMs.

Other minor comments and typos
page 2, second column "[...] N is the number of discrete grids. " -> should probably be grid-points.
page 8, second column "rensult" -> "result".
The font size used in most figures is too tiny.

This is Simon Olsson, FU Berlin.

Reviewer #2 (Remarks to the Author):

The authors propose to use Long Short-Term Memory (LSTM) Neural Networks as a tool to learn kinetic properties of complex molecular systems. I think it is an interesting idea, as it shows how an AI method can be used to analyze molecular trajectories, and might stimulate further ideas. However, I think the paper cannot be published at this stage because of two main problems:

1. The systems on which the method is applied are too simple to highlight its usefulness.
2. A theoretical and practical comparison with Markov state models, which are the standard approach used to learn molecular kinetics, is missing.

I elaborate more on these two issues below and I add a few other suggestions that the authors might want take into account.

As far as I understand, the introduced method takes as an input a trajectory binned on some observed variable. The examples are chosen so as to show what happens when the specific variable is not able to correctly describe the connectivity of the higher-dimensional underlying representation (with the exception of the simplest example of Fig. 2a). However, since the variable is binned first and then transformed in a one-hot encoding, the connectivity implied by the initial variable is not used at all. The only information used is the actual binning. This is not different from doing a cluster analysis on the low-dimensional representation provided by the chosen observable. So, this is not a proper way to make the "exercise difficult" so as to judge the merits of the method.

A challenging example in this sense would be one where the chosen low-dimensional representation is not able to distinguish two of the metastable structures. For instance, what happens if states B and C in Fig. 2c merge in a single value of x? This would be a difficult case where the correct dynamics could only be inferred by a hidden variable implicitly propagated by the LSTM. The LSTM would need to be thus trained to distinguish different instances of what is apparently the same state. To use the authors analogy, the LSTM would have to capture the different meaning of the same word in different contexts, which is exactly what this approach was designed for. This is however not apparent in the analyzed systems.

I think this issue is highly related with the missing detailed comparison with Markov state models (MSM). The authors fairly declare in the conclusion that "This work represents a first step in this direction, wherein we used AI, specifically LSTM flavor of recurrent neural networks, to perform kinetic reconstruction tasks that other methods could have also performed", referring to MSM. However, the

similarity is very high and deserves more than a quick mention. I argue here that a standard MSM could be described within an identical framework as a model that, given the time series of one-hot vectors s(t), infers a transition matrix by counting the observed transitions with a time lag. An MSM thus has a single parameter to be learned at this stage (the time lag), at variance with the many parameters that should be learned by the LSTM. A standard approach in MSMs is indeed to vary the time lag and select it such that transitions are Markovian. Which is the advantage of learning many parameters as they are needed in the LSTM framework? Is the presence of more parameters a possible source of overfitting? Does it improve the resulting kinetics in practice?

Another thing that is not well discussed in the manuscript is the importance of what is called smoothening (removal of ephemeral/spurious transitions) in SI. I can imagine that in the model of Fig. 2c one might end up in troubles if all A<->C transitions were detected as passing through B, as it happens in the low-dimensional representation x. In other words, there is a state along the transition between A and C that might be incorrectly merged to B by the observed variable. In an MSM, this problem would be solved by either (a) using a time lag large enough to statistically remove the weight of these spurious states in the transition count or (b) using core-set MSMs. I was expecting the introduced LSTM to be at least able to eliminate these spurious states automatically, based on the context. However, it looks like this is done at a pre-processing stage. Is this done manually by the authors? Do they evaluate the final performance of the model in order to decide how to smoothen the trajectory?

A merit of the current manuscript is that it gives a physical interpretation for the embedding layer. However, it is difficult to appreciate it without a theoretical and practical comparison with analogous tools widely used in the MSM community, such as projections on the eigenvectors of the transition matrix or state lumping.

I also found a couple of typos:

Page 8: "As a rensult" -> "As a result"

SI, page one: "As is typical in character-level language models? " There is a missing reference.

Response to reviewer 1

We thank this reviewer for their very encouraging report and for the excellent suggestions. We have performed additional simulations as suggested by this reviewer, and implemented their suggestions in the revised manuscript. In what follows, we have reproduced all the comments of this reviewer in italics, followed by our response in bold after asterisk. Any changes resulting due to the comments of this reviewer are highlighted in color blue in the revised manuscript.

Reviewer #1 (Remarks to the Author):

Review of "Learning Molecular Dynamics with Simple Language Model built upon Long Short-Term Memory Neural Network" by Tsai, Kuo, and Tiwary.

In their manuscript, the authors apply long short-term memory (LSTM) neural networks to analyze the trajectories of stochastic dynamics. Data-driven methods, and in particular machine learning/AI methods, in this area have a broad scope of application, including the analysis of experimental and simulation data to build an understanding of the underlying physics, chemistry, or biology of complex systems.

There is a long history of literature in this field, in particular in statistical physics, and more recently also in applied machine learning. Particularly successful methods include Master equation-based methods for conformational dynamics / Markov state Models, Hidden Markov models, time-lagged Independent Component analysis. More recently, AI/ML methods such as Dynamic Graphical Models and VAMP-derived methods (Variational approach for learning Markov Processes), including VAMPnets, show great promise.

The application to LSTM to dynamical systems in the natural sciences is sound, as this architecture is tailor-made to tackle sequential data. What is particularly intriguing about LSTMs is their ability to maintain information beyond the previous state to predict the current state. In effect, this means that we can relax the assumption of first-order Markovianity of the observed dynamics, that many current methods invoke. However, it remains unclear whether this relaxation may be beneficial from a practical perspective, in particular when the only dominant dynamical modes are of interest. Further, an interpretation of the LSTM from physical point-of-view is currently lacking in the best of this readers' knowledge. This gap may be a cause of concern for members of our community who are skeptical of 'black-box' methods.

While the use of LSTMs to dynamic problems in the natural sciences appears like a straightforward endeavor, the authors go well beyond applying pre-existing AI-methodology. In particular, the authors make the following significant contributions and explicitly address some critical gaps in our understanding:

- They show that the minimization of a cross-entropy loss used to train LSTMs of natural

languages corresponds to matching the cross-entropy of true trajectory path and the modeled trajectory path. In particular, they show that the path entropy of the true dynamical process bounds this cross-entropy. 'True' here refers to the process observed in data used to train the model.

- They illustrate how the learned embedding space (A linear transformation of a spatial encoding of a time-point) enter into expressions of the transition probability (under an assumption of a stationary first-order Markovianity assumption) and may capture the average commute time between two points.

- Through numerical experiments on several tractable model systems, the authors further illustrate how the LSTM accurately predicts key thermodynamic and kinetic properties of the underlying systems.

I find that the manuscript should be published. The study addresses a highly interesting topic of interest to a broad community of researchers. Overall, the study gives the impression to be well-planned and rigorously executed, and presents theoretical and computational results in a well-written and visually appropriate manner.

I only have a handful of minor suggestions that may help underline the significance of the points made in the manuscript to a broader audience:
Please compare all the results from the LSTMs to the current state of the art methods using a similar state encoding (a discretization of a sub-optimal 1D reaction coordinate). Either Markov state models or Hidden Markov models.

*** We have added a subsection with a comparison of MSM and HMM, in which we briefly go through the advantages of using LSTM relative to MSM and HMM by providing detailed analyses for our various same model systems. In order to not distract the readers, all analyses have been put in the supplementary materials except for the linear 3-state case which is provided in the main text itself.**

I think this point is particularly important as the authors themselves state in the conclusion that these methods can perform a similar task. Still, they later state that they expect LSTM may, in general, outperform traditional methods. Consequently, having traditional methods as baselines would be a helpful first step towards substantiating this statement.
It would be helpful to include an example that illustrates the analysis of an experimental time-series, or simulation data of a protein or nucleic acid system, fx from protein-folding. And again, compare these analyses state of the art analysis techniques. An example could be single-molecule FRET trajectories analyzed via HMMs.

*** This is a wonderful suggestion. We contacted the group of Michael Woodside at the University of Alberta, who kindly provided us with a single-molecule FRET trajectory (details in main text). We analyzed this trajectory using LSTM, MSM, and HMM. The results using LSTM have now been shown in an independent subsection regarding experimental details, while the HMM, MSM details are**

provided in SecIV E. We find here that relative to MSM and HMM, LSTM has a better agreement in a small commitment time region.

Other minor comments and typos
page 2, second column "[...] N is the number of discrete grids. " -> should probably be grid-points.
page 8, second column "rensult" -> "result".

*** These typos have been corrected.**

The font size used in most figures is too tiny.

*** The font size used in all figures has been increased.**

Response to reviewer 2

We thank this reviewer for their encouraging report and for the excellent suggestions. We have performed additional simulations as suggested by this reviewer, and implemented their suggestions in the revised manuscript as well as the extensively modified SI. In what follows, we have reproduced all the comments of this reviewer in italics, followed by our response in bold after asterisk. Any changes resulting due to the comments of this reviewer are highlighted in color red in the revised manuscript.

Reviewer #2 (Remarks to the Author):

The authors propose to use Long Short-Term Memory (LSTM) Neural Networks as a tool to learn kinetic properties of complex molecular systems. I think it is an interesting idea, as it shows how an AI method can be used to analyze molecular trajectories, and might stimulate further ideas. However, I think the paper cannot be published at this stage because of two main problems:

1. The systems on which the method is applied are too simple to highlight its usefulness.
2. A theoretical and practical comparison with Markov state models, which are the standard approach used to learn molecular kinetics, is missing.

I elaborate more on these two issues below and I add a few other suggestions that the authors might want take into account.

*** We appreciate the reviewer's concern. In order to allay both #1 and #2 concerns above, we contacted the group of Michael Woodside at the University of Alberta, who kindly provided us with a single molecule force spectroscopy trajectory for a riboswitch pulling experiment (details in main text). We analyzed this trajectory using our model as well as markov state models (MSM), and hidden markov models (HMM). We also analyzed our other systems in this work using MSM and HMM. As can been seen in the analysis of linear 3-state model system by MSM and HMM provided in the modified main text and the SI, even for this simple system it is difficult enough for them to find the exact kinetics from the binned trajectory.**

As far as I understand, the introduced method takes as an input a trajectory binned on some observed variable. The examples are chosen so as to show what happens when the specific variable is not able to correctly describe the connectivity of the higher-dimensional underlying representation (with the exception of the simplest example of Fig. 2a). However, since the variable is binned first and then transformed in a one-hot encoding, the connectivity implied by the initial variable is not used at all. The only information used is the actual binning. This is not different from doing a cluster analysis on the low-dimensional representation provided by the chosen observable. So, this is

not a proper way to make the "exercise difficult" so as to judge the merits of the method.

*** The reviewer is correct in saying "This is not different from doing a cluster analysis on the low-dimensional representation provided by the chosen observable."; however, we want to emphasize that we always clustered the low-dimensional representation with equally spaced bins and very similar LSTM pararameters even when learning from the trajectories of alanine dipeptide and single molecule force spectroscopy experiment - two very disparate systems. That is to say, the LSTM does not require users to know how to do an appropriate clustering. Without an appropriate clustering, it is easy to make the time series behave non-Markovian, which is the reason why MSM and HMM fail to fit the kinetics of the experimental data.**

A challenging example in this sense would be one where the chosen low-dimensional representation is not able to distinguish two of the metastable structures. For instance, what happens if states B and C in Fig. 2c merge in a single value of x? This would be a difficult case where the correct dynamics could only be inferred by a hidden variable implicitly propagated by the LSTM. The LSTM would need to be thus trained to distinguish different instances of what is apparently the same state. To use the authors analogy, the LSTM would have to capture the different meaning of the same word in different contexts, which is exactly what this approach was designed for.

*** This is an ambitious ask, and perhaps achievable in the use of molecular dynamics trajectories as well. We sincerely request the reviewer to let us leave this for future work as believe the current work itself marks a step ahead in the use and understanding of LSTM (through its connections with path entropy and embedding layer interpretations that we derive here). In future, building on this base, we will carefully push LSTM to even more ambitious applications including the one suggested by this reviewer.**

This is however not apparent in the analyzed systems.

I think this issue is highly related with the missing detailed comparison with Markov state models (MSM). The authors fairly declare in the conclusion that "This work represents a first step in this direction, wherein we used AI, specifically LSTM flavor of recurrent neural networks, to perform kinetic reconstruction tasks that other methods could have also performed", referring to MSM. However, the similarity is very high and deserves more than a quick mention.

*** We completely agree with the reviewer and have taken their suggestion into full consideration. We have added a subsection with a comparison of MSM and HMM, in which we briefly go through the advantages of using LSTM than MSM and HMM. We also provide a similar analysis of MSM and HMM using the same trajectories for different systems. Some of these new results are given in the main text while many other detailed ones are provided in the SI due to size considerations.**

I argue here that a standard MSM could be described within an identical framework as a model that, given the time series of one-hot vectors s(t), infers a transition matrix by counting the observed transitions with a time lag. An MSM thus has a single parameter to be learned at this stage (the time lag), at variance with the many parameters that should be learned by the LSTM. A standard approach in MSMs is indeed to vary the time lag and select it such that transitions are Markovian. Which is the advantage of learning many parameters as they are needed in the LSTM framework?

*** The advantages of having more parameters in LSTM is that the LSTM can be used without pre-selected lag time and an appropriate clustering of the low-dimensional representation of the chosen observable in order to capture the kinetics. In addition, LSTM network does not require the trajectory to abide by Markov property. As can be seen from the discussion related to the previous comment of the reviewer, the LSTM is much more robust at capturing even short-time kinetics than the other methods.**

Is the presence of more parameters a possible source of overfitting?

*** This is an excellent point and indeed always a concern in multi-parameter models and especially neural networks. To avoid overfitting, we carefully look at the validation loss and training loss during training of the network. Once the validation loss becomes slightly larger than the training loss, it is considered traditionally as the onset of overfitting. In all of the independent trainings of the LSTM network, we choose to fix the training epoch when the validation losses are always approximately equal to the training loss. In order to show the robustness, the results were averaged over several independent trainings of the same training epoch.**

Does it improve the resulting kinetics in practice?

*** Yes, please see the comparison with MSM and HMM in Fig. 6 and 7.**

Another thing that is not well discussed in the manuscript is the importance of what is called smoothening (removal of ephemeral/spurious transitions) in SI. I can imagine that in the model of Fig. 2c one might end up in troubles if all A<->C transitions were detected as passing through B, as it happens in the low-dimensional representation x. In other words, there is a state along the transition between A and C that might be incorrectly merged to B by the observed variable. In an MSM, this problem would be solved by either (a) using a time lag large enough to statistically remove the weight of these spurious states in the transition count or (b) using core-set MSMs. I was expecting the introduced LSTM to be at least able to eliminate these spurious states automatically, based on the context. However, it looks like this is done at a pre-processing stage. Is this done manually by the authors?

*** We thank the reviewer for raising this concern, and explain our reasoning/methods further here as well as through modified main text. Please also see related discussion for the next point by this reviewer. First of all, for alanine dipeptide and the force spectroscopy data we did not even perform smoothening to validate our rationale. In the benchmark systems, we performed the smoothening to avoid the spurious states because we do not claim here the LSTM can distinguish two different states when it happens the two states are overlapped on the low-dimensional representation. However, there is a way for LSTM to capture the kinetics without doing smoothening, which is to increase the precision in state space. In other words, if we use a larger number of bins for benchmark systems, we are still able to avoid the spurious states without smoothening data. Though at the same time, it makes the embedding space harder to understand. This can be seen in the test on alanine dipeptide because we did not smoothen the trajectory there. For the newly added force spectroscopy trajectory, we again smoothen the data because it is hard to even see the three states from the raw data.**

The key take away here and in the next point is that while smoothening helps, we do not have to hand-tune how to implement it.

Do they evaluate the final performance of the model in order to decide how to smoothen the trajectory?

*** This is an excellent point: the final performance of the model is not taken into account to decide the smoothening protocol. The two are independent of each other. We explain why this works through intuitive arguments below.**

LSTM always fits the Boltzmann statistics and kinetics. Without smoothening, the connectivity would be wrong which is simply because the kinetics of the binned trajectory is wrong: Take the 4-state model system as an example. When projecting on x-axis, there are no spurious states existing between A and D since they are far enough on x-axis, while there are many between B and C. The states B and C are too close such that there is a high chance of miscounting the spurious recrossings between B and C as the transitions between them. As we've mentioned, if choosing a large number of bins for spatial discretization, we are still able to obtain correct kinetics without smoothening. Showing results of smoothening is a way to simplify the result. When applying LSTM to alanine dipeptide and experimental data, we do not smoothen the trajectory.

A merit of the current manuscript is that it gives a physical interpretation for the embedding layer. However, it is difficult to appreciate it without a theoretical and practical comparison with analogous tools widely used in the MSM community, such as projections on the eigenvectors of the transition matrix or state lumping.

*** We thank the reviewer for appreciating this merit, and hope the manuscript is deemed worthy of publication in light of multiple new comparisons and analyses**

we have now provided.

I also found a couple of typos:

Page 8: "As a rensult" -> "As a result"
SI, page one: "As is typical in character-level language models? " There is a missing reference.

*** The typos and incorrect reference have been corrected.**

Reviewer #1 (Remarks to the Author):

I thank the authors for carefully considering my input in the previous review round. I have no further comments.

Reviewer #2 (Remarks to the Author):

The authors replied to my points. I like the additional example on the three state riboswitch, although I think there are still minor points to be fixed before the paper is publishable.

The authors declare that their method, at this stage, is not able to identify the contextual meaning of an observation, since the authors were not able to include an example where the same observation (bin) corresponds to two different metastable states. I am fine with this, but it should be written in a transparent way in the text. In particular, this limitation should be mentioned in the abstract, since that's the place where the original LSTM aim is mentioned (i.e., representing the contextual meaning) and briefly discussed in the conclusions. This will make the scope of the method clearer, and will also make it clear where new efforts (by this or other groups) should be directed.

A detail that should be clarified is if the binning used in the MSM is identical to the one used in the LSTM. I assume so, otherwise the comparison would not be meaningful, but I did not find this information. In other words: is the same one-hot encoding fed to MSM, HMM, and LSTM?

We thank the reviewer for appreciating the additional work we did and for the final suggestions. In what follows, we have reproduced all the comments of this reviewer in italics, followed by our response in bold after asterisk. Any changes resulting due to the comments of the reviewer are highlighted in color blue in the revised manuscript.

Reviewer #2 (Remarks to the Author):

The authors replied to my points. I like the additional example on the three state riboswitch, although I think there are still minor points to be fixed before the paper is publishable.

The authors declare that their method, at this stage, is not able to identify the contextual meaning of an observation, since the authors were not able to include an example where the same observation (bin) corresponds to two different metastable states. I am fine with this, but it should be written in a transparent way in the text. In particular, this limitation should be mentioned in the abstract, since that's the place where the original LSTM aim is mentioned (i.e., representing the contextual meaning) and briefly discussed in the conclusions. This will make the scope of the method clearer, and will also make it clear where new efforts (by this or other groups) should be directed.

*** We appreciate the reviewer's concern. In the abstract, we have replaced "The embedding layer, introduced originally for representing the contextual meaning …" by "The embedding layer, instead of representing the contextual meaning …". We have also added a discussion in the conclusion part about using the embedding layer to distinguish different metastable states with the same observation in future work.**

A detail that should be clarified is if the binning used in the MSM is identical to the one used in the LSTM. I assume so, otherwise the comparison would not be meaningful, but I did not find this information. In other words: is the same one-hot encoding fed to MSM, HMM, and LSTM?

*** The binning (and one-hot encoding) used in the MSM/HMM is identical to the one used in the LSTM. We have clarified this missing point in the main text.**
